# Regulation of the lncRNA NEAT1 by p53‐ΔNp63 crosstalk modulates the DNA damage response and therapeutic efficacy in HNSCC

**DOI:** 10.1002/1878-0261.70331

**Published:** 2026-09-07

**Authors:** Sara De Domenico, Veronica La Banca, Silvia D'Amico, Stefano Scalera, Sara Nicolai, Angelo Peschiaroli

**Affiliations:** ^1^ Institute of Translational Pharmacology (IFT), CNR Rome Italy; ^2^ Clinical Trial Center, Biostatistics and Bioinformatics Unit, Department of Research, Diagnosis and Innovative Technologies IRCCS Regina Elena National Cancer Institute Rome Italy

**Keywords:** DNA damage, histone deacetylase, HNSCC, NEAT1, transcription factors

## Abstract

Head and neck squamous cell carcinomas (HNSCCs) are characterized by recurrent genetic alterations, including the inactivation of the tumor suppressor *TP53* gene and dysregulation of the *TP63* gene. The *TP63* gene encodes multiple isoforms, among which the N‐terminal truncated isoform ΔNp63 is fundamental for the integrity of stratified epithelial tissues. We previously demonstrated that ΔNp63 represses the expression of the lncRNA NEAT1. Here, we investigated the functional crosstalk between p53 and ΔNp63 in modulating NEAT1 expression following genotoxic stress. We found that upon genotoxic insults, p53 activation and the concomitant downregulation of ΔNp63 promote NEAT1 transcription. In p53‐proficient HNSCC cells, NEAT1 targeting leads to increased DNA damage, highlighting its potential role in maintaining genomic stability and facilitating efficient DNA repair. Importantly, we showed that histone deacetylase inhibitors (HDACis) upregulate NEAT1 expression independently of p53, and NEAT1 silencing enhances HDACis‐induced DNA damage. Overall, our findings establish NEAT1 as an early regulator of the DNA damage response in HNSCCs and suggest that combining NEAT1 targeting with HDAC inhibition may potentiate therapeutic efficacy, particularly in *TP53*‐mutant HNSCCs.

## Graphical abstract

In head and neck squamous cell carcinoma (HNSCC) p53 and p63 exert opposite roles on the transcription regulation of the lncRNA NEAT1. Under basal conditions, p53 levels are low and p63 represses NEAT1 expression. Upon genotoxic stress, p53 is rapidly induced, displacing p63 from the NEAT1 promoter leading to NEAT1 transcriptional activation and subsequent induction of the DNA damage response (DDR) pathway. The figure was drawn using images created in Microsoft 365 PowerPoint (CNR license).

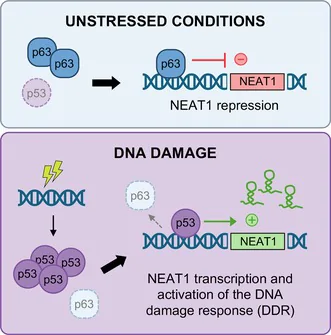

**Abbreviations**
ATM: ataxia telangiectasia mutated protein
ChIP: chromatin immunoprecipitation assay
DDR: DNA damage response
FISH: Fluorescence *In Situ* Hybridization
HDAC1: histone deacetylases 1
HDAC2: histone deacetylases 2
HNSCC: squamous cell carcinoma of head and neck
NEAT1: Nuclear Enriched Abundant Transcript 1
PARP: Poly (ADP‐ribose) polymerase
SCC: squamous cell carcinoma
UVB: ultraviolet B radiation

## 1 Introduction

Head and neck squamous cell carcinomas (HNSCCs) are among the most common epithelial malignancies worldwide. They arise from the stratified squamous mucosal epithelium of the oral cavity, larynx, and pharynx and are characterized by marked molecular heterogeneity, frequent recurrence, and poor clinical outcome [1]. HNSCCs' development is driven by the interplay between environmental risk factors, including tobacco smoking, alcohol consumption, viral infections, and recurrent genetic alterations [2]. At the molecular level, HNSCCs display widespread deregulation of pathways involved in genomic stability, as highlighted by frequent alterations affecting members of the p53 family. Indeed, *TP53* is mutated in most HNSCCs, and its loss contributes to genomic instability, therapeutic resistance, and disease progression [3, 4]. In parallel, amplification and overexpression of *TP63*, particularly of the N‐terminal truncated isoform ΔNp63, are common events in squamous cell carcinomas (SCC) [5]. ΔNp63 plays a central role in maintaining the proliferative potential of stratified epithelia and acts as a key regulator of squamous tumorigenesis through transcriptional programs controlling stemness, proliferation, and differentiation [6, 7, 8, 9, 10]. Importantly, p53 and ΔNp63 exhibit extensive functional interplay and often exert antagonistic effects on common transcriptional targets involved in cell cycle regulation, differentiation, and genome surveillance [11]. However, the molecular mechanisms through which this transcriptional crosstalk shapes the cellular response to genotoxic stress in HNSCC remain poorly understood.

Long noncoding RNAs (lncRNAs) are a class of untranslated transcripts that constitute a substantial fraction of the mammalian transcriptome. Through interactions with DNA, RNA, and RNA‐binding proteins, lncRNAs regulate multiple layers of gene expression, including chromatin organization, transcription, RNA processing, and translation [12, 13]. Among lncRNAs, NEAT1 (Nuclear Enriched Abundant Transcript 1) has attracted considerable attention because of its essential role in the assembly of paraspeckles, dynamic membrane‐less nuclear bodies implicated in transcriptional and post‐transcriptional adaptation to cellular perturbations. The *NEAT1* locus generates two overlapping isoforms with distinct expression patterns: the ubiquitously expressed short isoform NEAT1_1 (3.7 kb) and the long isoform NEAT1_2 (22.7 kb), whose expression is more restricted and strongly induced under stress conditions. Notably, only NEAT1_2 is indispensable for paraspeckles formation [14, 15].

NEAT1 has emerged as an important regulator of several biological processes controlling cell fate, including proliferation, differentiation, and responses to genotoxic stress [16, 17]. Its expression is tightly regulated by multiple signaling pathways and transcription factors [16]. Accumulating evidence indicates that NEAT1 contributes to the maintenance of genomic integrity in response to DNA damage and replication stress [18, 19]. Mechanistically, NEAT1 has been identified as a direct transcriptional target of p53 within the DNA damage response (DDR). In this context, p53‐mediated induction of NEAT1 promotes paraspeckles assembly and supports adaptive gene expression programs required to prevent excessive accumulation of DNA damage [18, 20, 21]. Consistently, loss of NEAT1 impairs stress adaptation and reduces cell fitness under genotoxic conditions [18]. Aberrant NEAT1 expression has been reported in multiple human malignancies, including squamous tumors, where it contributes to tumor progression, invasive potential, and therapeutic resistance through the modulation of stress‐adaptive transcriptional programs [22, 23].

More recently, we identified a novel mechanism of NEAT1 regulation mediated by ΔNp63, a master transcription factor of epithelial identity. In proliferating human keratinocytes, ΔNp63 represses NEAT1 transcription by recruiting HDAC1/2 to the *NEAT1* promoter, thereby establishing a transcriptionally repressive chromatin environment [24]. Upon induction of keratinocyte differentiation, the physiological decline in ΔNp63 levels relieves this repression, resulting in NEAT1 accumulation and paraspeckles formation. In line with this mechanism, pharmacological inhibition of HDAC activity also promotes NEAT1 expression [24].

Notably, p53 and ΔNp63 recognize the same DNA‐binding region within the *NEAT1* promoter, suggesting a competitive regulatory mechanism controlling NEAT1 transcription [18, 24]. Given its dual and context‐dependent nature, we aimed to elucidate how the transcriptional interplay between p53 and ΔNp63 regulates NEAT1 expression in response to genotoxic stress in normal epithelial and squamous carcinoma cells. We investigated how this regulatory axis influences DNA repair efficiency and cell survival, and how its disruption may contribute to tumor initiation and progression. Our findings identify NEAT1 as an early regulator of stress adaptation downstream of the p53–ΔNp63 crosstalk and demonstrate that its targeting enhances the cytotoxic effects of HDAC inhibitors in *TP53*‐mutant HNSCC cells, suggesting a potential combinatorial therapeutic strategy for these tumors.

## 2 Material and methods

### 2.1 Cell lines

Ker‐CT cells (hTERT‐immortalized keratinocyte, ATCC CRL‐4048, RRID:CVCL_S877) were cultured in EpiLife medium with addition of human keratinocyte growth supplements (HKGS, Life Technologies, Grand Island, USA); HN30 cells (Pharyngeal squamous cell carcinoma, Runtogen, RWT‐1241, RRID:CVCL_5525) were cultured in DMEM's medium (Gibco, Invitrogen); A253 cells (submaxillary salivary gland carcinoma, male, ATCC HTB‐41, RRID:CVCL_1060) were cultured in McCoy's medium (Gibco, Grand Island, USA; Invitrogen, Carlsbad, USA); FaDu (Pharynx squamous cell carcinoma, male, ATCC HTB‐43, RRID:CVCL_1218) were grown in EMEM (Lonza, Basel, Switzerland); Detroit‐562 (Pharynx squamous cell carcinoma, female, ATCC CCL‐138, RRID:CVCL_1171) were grown in EMEM; SCC7 cells (Mouse squamous cell carcinoma, RRID:CVCL_V412) were grown in RPMI; MOC1 (Squamous cell carcinoma of the mouse oral cavity, Kerafast EWL001‐FP, RRID:CVCL_ZD32) were grown in Iscove's Modified Dulbecco's Medium (IMDM)/Nutrient Mixture F‐12 (2 : 1) (Cytiva, Uppsala, Sweden; SH30228.01 and SH30026.01, respectively), supplemented with 5 μg/mL insulin, 40 ng/mL hydrocortisone, and 5 ng/mL EGF. HN30, A253, FaDu, Detroit‐562, and SCC7 growth media were supplemented with 10% fetal bovine serum (FBS), while MOC1 growth medium was supplemented with 5% FBS. All cell lines were supplemented with 100 μg/mL penicillin/streptomycin (Pen/Strep) (Gibco, Invitrogen). Cell lines were purchased from the indicated suppliers and cultured at early passages at 37 °C with 5% CO_2_. SCC7 were a kind gift from Reinhard Zeidler of the Helmholtz Zentrum München (HMGU). Cells were routinely tested for mycoplasma contamination by MycoAlert mycoplasma detection kit (LONZA LT07‐418) and all experiments were performed with mycoplasma‐free cells. FaDu, A253, HN30, and Detroit‐562 were authenticated by PCR‐locus‐technology in 2026 (Eurofins, Ebersberg, Germany).

### 2.2 Treatments and transfection

The histone deacetylase inhibitors givinostat (Sigma‐Aldrich, St. Louis, USA; #SML1772), entinostat (TargetMol, Boston, USA; #T6233), and panobinostat (TargetMol, #T2383) were added to the culture medium at the indicated concentrations for 18 h. Doxorubicin (Sigma‐Aldrich, #D5220) was administered at final concentrations of 1 μm or 0.2 μm for 18 h. For UVB exposure, culture dishes were placed without medium in a closed chamber equipped with a UVB light source, delivering a total dose of 30 mJ, and cells were analyzed at the indicated time points. For siRNA oligos and Antisense LNA GapmeRs transfection, cells were transfected using the Lipofectamine RNAiMAX transfection reagent (Invitrogen) according to the company's protocols. For NEAT1 silencing, a mixture of LNA GapmeRs (LNA‐NEAT1#4, #6, and #7) was used. All siRNAs and LNA GapmeRs used are listed in Table 1.

**Table 1:** List of reagents.

Antibodies	Supplier	Catalog	Dilution
Rabbit monoclonal anti‐p63α (clone D2K8X)	Cell Signaling Tech	#13109	1 : 1000
Rabbit polyclonal anti‐PARP	Cell Signaling Tech	#9542	1 : 1000
Rabbit monoclonal anti‐Cleaved Caspase‐3 (clone 5A1E)	Cell Signaling Tech	#9664	1 : 1000
Rabbit monoclonal anti‐p21	Cell Signaling Tech	#2946	1 : 1000
Rabbit monoclonal anti‐Phospho‐p53 (Ser15)	Cell Signaling Tech	#82530	1 : 1000
Mouse monoclonal anti‐p53	Santa Cruz Biotech	#sc‐126	1 : 1000
Mouse monoclonal anti‐Phospho‐H2AX (Ser139) (clone JBW301)	Merck Millipore	#16193	1 : 1000
Rabbit monoclonal anti‐β‐tubulin	Santa Cruz Biotech	#2146	1 : 5000
Mouse monoclonal anti‐β‐actin (clone AC‐15)	Sigma‐Aldrich	#A5441	1 : 50000
Mouse monoclonal anti‐GAPDH	Sigma‐Aldrich	#G8795	1 : 5000
Moumouse monoclonal anti‐vinculin	Sigma‐Aldrich	#V9131	1 : 50000

siRNA and antisense LNA GapmeRs	Sequence
sip53	5′‐GUAAUCUACUGGGACGGAA‐3′
sip63#2	5′‐CAGGUUGGCACUGAAUUCA‐3′
siScr	negative ctrl #1 SIC001 Sigma‐Aldrich
LNA‐NEAT1#4	5′‐TAAGCACTTTGGAAAG‐3′
LNA‐NEAT1#6	5′‐CAAGGAAAGTCATCGC‐3′
LNA‐NEAT1#7	5′‐AATAGACGTGAGTGGA‐3′
LNA‐Control	5′‐AACACGTCTATACGC‐3′

Primers for qPCR	Sequence
NEAT1 Fwd	5′‐TGGCTAGCTCAGGGCTTCAG‐3′
NEAT1 Rev	5′‐TCTCCTTGCCAAGCTTCCTTC‐3′
NEAT1_2 Fwd	5′‐GGCCAGAGCTTTGTTGCTTC‐3′
NEAT1_2 Rev	5′‐GGTGCGGGCACTTACTTACT‐3′
∆Np63 Fwd	5′‐GAAGAAAGGACAGCAGCATTG‐3′
∆Np63 Rev	5′‐ GGGACTGGTGGACGAGGAG‐3′
p21 Fwd	5′‐TGAGCGATGGAACTTCGA‐3′
p21 Rev	5′‐ACAAGACAGTGACAGGTCC‐3′
GAPDH Fwd	5′‐GTCTCCTCTGACTTCAACAGCG‐3′
GAPDH Rev	5′‐ACCACCCTGTTGCTGTAGCCAA‐3′
TBP Fwd	5′‐TGTATCCACAGTGAATCTTGGTTG‐3′
TBP Rev	5′‐GGTTCGTGGCTCTCTTATCCTC‐3′

Primers for ChIP	Sequence
NEAT1 locus Fwd	5′‐GAAACAGGGCTAAGCAGGCG‐3′
NEAT1 locus Rev	5′‐GCTTCTCGGAAAACTGGTGAC‐3′

### 2.3 RNA isolation and RT‐qPCR

Total RNA was isolated using the RNeasy mini kit (Qiagen, Hilden, Germany) following the company's recommendations, quantified by a NanoDrop Spectrophotometer (Thermo Scientific, Waltham, USA) and retrotranscribed by SensiFast cDNA Synthesis (Bioline, London, UK). Real‐time PCR was performed with the PowerUp SYBR‐Green PCR Master Mix (Thermo Scientific, cat. num. A25742) in the QuantStudio 1 Real‐Time PCR Systems (Applied Biosystems, Waltham, USA). The expression of each gene was defined from the threshold cycle (Ct), and the relative expression levels were calculated using the 2^−ΔΔCt^ method. The primers utilized for RT‐qPCR are shown in Table 1.

### 2.4 Protein extraction and immunoblotting analyses

Immunoblot analysis was performed as previously described [24]. Briefly, cell extracts were obtained by lysing cell pellets with Triton buffer (50 mm Tris/HCl pH 7.5, 250 mm NaCl, 50 mm NaF, 1 mm EDTA pH 8, 0.1% Triton) or RIPA buffer (50 mm Tris/HCl pH 8, 150 mm NaCl, 1% NP‐40, 0.5% DOC, 0.1% SDS, 1 mm EDTA pH 8), supplemented with protease inhibitors (Roche, Basel, Switzerland), DTT, PMSF, and sodium orthovanadate (NEB). Proteins were separated by SDS/PAGE, transferred onto PVDF membranes, and blocked with PBS‐T (phosphate‐buffered saline and 0.1% Tween‐20) containing 5% nonfat dry milk for 1 h at room temperature (RT) or with EveryBlot Blocking Buffer (Bio‐Rad #12010020). The incubation with primary antibodies was performed for 2 h at RT or overnight at 4 °C, followed by 1‐h incubation with the appropriate horseradish peroxidase (HRP)‐conjugated secondary antibody (Bio‐Rad anti‐mouse cat. num. 170‐5047, dilution: 1 : 10000; Bio‐Rad anti‐rabbit cat. num. 170‐6515, dilution: 1 : 10000). Detection was performed with ECL chemiluminescence kit (Perkin‐Elmer, Waltham, Massachusetts, USA) and the signal was acquired with Uvitec Alliance Atom (Uvitec, Cambridge, UK). The complete list of the antibodies used is reported in Table 1. Uncropped images related to the blots are shown in Figs S12 and S13.

### 2.5 Cell cycle analysis

Cells were resuspended in 500 μL of PBS and fixed by adding 500 μL of methanol/acetone (4 : 1, v/v). Fixed cells were then washed and incubated in 50 μL of RNase A solution (100 μg/mL) for 15 min at RT. Cells were subsequently stained with 300 μL of propidium iodide (PI) solution (50 μg/mL in PBS) or DAPI (1 μg/mL in PBS), at RT for 30 min in the dark. Flow cytometry analysis was carried out using a cytoflex S flow cytometer (Beckman Coulter Life Sciences, Brea, USA), acquiring 20 000 events per sample. Cell cycle distribution was determined using FlowJo 10.10.1 (Becton Dickinson & Company (BD), Franklin Lakes, USA).

### 2.6 Colony formation assay

Following the treatments described above, cells were seeded in 6‐well plates at low density (800 cells/well) and incubated for two weeks to allow colony formation. Colonies were fixed with 4% formaldehyde in PBS, stained with 0.1% crystal violet (Sigma‐Aldrich), and then washed with water and dried at room temperature.

### 2.7 Immunofluorescence

Cells grown on coverslips were fixed with buffered formalin for 10 min at RT, then permeabilized by incubation in PBS 0.25% Triton X‐100 (Sigma‐Aldrich, #T8787) for 20 min and IF staining was performed as follows: 1 h blocking in 5% goat serum (Gibco) in PBS at RT; 2 h primary antibody incubation at RT; 1 h secondary antibody and DAPI (4′,6‐diamidino‐2‐phenylindole) incubation at RT. The following antibodies were used: mouse monoclonal anti‐Phospho‐histone H2AX (Ser139) (Merck Millipore #16193, Burlington, USA; clone JBW301, dilution 1:300), goat anti‐mouse 488 (Life Technologies, A28175, dilution: 1 : 1000). Images were acquired using a Leica Stellaris 8 confocal microscope. Cells displaying more than 5 γH2AX nuclear foci were considered positive, and the percentage of γH2AX‐positive cells was calculated on the total number of cells. For each condition, a minimum of 200 cells was analyzed.

### 2.8 RNA fluorescence *in situ* hybridization

RNA FISH experiments were performed using Stellaris® RNA FISH kit (Biosearch Technologies), following the manufacturer's recommendations. Ker‐Ct cells were cultured on coverslips and were fixed in 4% paraformaldehyde for 10 min and then permeabilized with 0.1% Triton X‐100 for 10 min. Incubation with the human NEAT1 probe with Quasar 570 Dye (Biosearch Technologies, Inc.) was performed for 16 h at 37 °C in a dark humid chamber. 1 μg/mL 4′,6‐diamidino‐2‐phenylindole (DAPI; Sigma) was added to the mix for nuclear DNA staining. Coverslips are placed on a microscope slide with ProLong Gold Antifade (Thermo Fisher Scientific) and images acquired on a confocal microscope Leica Stellaris 8. For each condition, a minimum of 200 cells were analyzed.

### 2.9 Chromatin immunoprecipitation (ChIP) assay

Ker‐CT cells treated with doxorubicin were used for ChIP assay. Cells were collected and fixed in 1% formaldehyde. After nuclei lysis, chromatin was sonicated into 200‐ to 500‐bp fragments using a Diagenode Bioruptor. The chromatin immunoprecipitation was performed with a rabbit monoclonal anti‐p63‐α antibody (Cell Signaling Technology #13109, Danvers, USA; clone D2K8X), mouse monoclonal anti‐p53 (Santa Cruz Biotechnology #sc‐126, Dallas, USA; dilution 1 : 1000), or unspecific immunoglobulin G (IgG) mouse and rabbit (Invitrogen) using the MAGnify Chromatin Immunoprecipitation System (Invitrogen, #492024). The primers used for the amplification of the *NEAT1* promoter region containing p63 and p53 binding sites are reported in Table 1.

Percent of Input was calculated with the following equation:

$$\%\text{Input}=2^{\mathrm{Ct}\left(\text{adjusted Input}\right)–\mathrm{Ct}\left(\mathrm{IP}\right)},$$

where Ct(adjusted Input) = Ct(Input) – log_2_(dilution factor).

### 2.10 Comet assay

DNA damage was assessed using the CometAssay Kit (Trevigen, Gaithersburg, USA; Cat. #4250‐050‐K) according to the company's instructions. Cells (1 × 10^5^/mL) were mixed with the LMAgarose at 37 °C and layered onto CometSlides™. After solidification at 4 °C, slides were incubated in lysis solution (4 °C, 30–60 min) and subsequently subjected to alkaline unwinding (200 mm NaOH, 1 mm EDTA, pH > 13) for 30 min at RT in the dark. Electrophoresis was then performed at 21 V for 30 min in alkaline electrophoresis buffer. Slides were washed in distilled water, fixed in 70% ethanol, dried at 37 °C, and stained with SYBR Gold (Thermo Fisher Scientific #S11494) for 30 min at RT in the dark. Comets were acquired on a confocal microscope Leica Stellaris 8 (excitation/emission 496/522 nm), with a minimum of 1.000 comets analyzed per condition. The comet assay parameters, %DNA in tail and Olive Tail Moment, were extracted using an opencomet v1.3.1 plugin in ImageJ. Specifically, % DNA in tail was defined as the proportion of total DNA in the comet tail, while the Olive Tail Moment was used as an additional sensitive index of DNA damage, combining the fraction of DNA in the tail with the distance between the centers of the head and the tail. These parameters were calculated as follows:

$$\%\mathrm{DNA}\;\text{in tail}=\begin{array}{l} {}[\text{tail intensity}/(\text{head intensity}\\ +\;\text{tail intensity})]\times 100. \end{array}$$

$$\text{Olive Tail Moment}=\begin{array}{l} (\text{Mean position of tail}\\ -\;\text{Mean position of head})\\ \times \%\mathrm{DNA}\;\text{in Tail} \end{array}$$

Differences in comet assay parameters were assessed using the Mann–Whitney statistical test in graphpad Prism (v10.2.1).

### 2.11 Bioinformatic analysis

Gene expression data for bioinformatic analysis were downloaded from The Cancer Genome Atlas (TCGA), including HNSCC (522 tumors, 44 normal), LUSC (501 tumors, 51 normal), and ESCA (96 tumors, 13 normal). Single‐cell RNA sequencing data were obtained from the publicly available GSE181919 dataset, comprising normal oral mucosa, primary HNSCC, and metastatic lymph node samples. Downstream analyses were performed in R using Seurat v5 [25]. Cell annotations provided by the original study were retained throughout the analysis. NEAT1 expression was quantified using normalized log1p‐transformed UMI counts. UMAP embeddings were generated from the processed Seurat object and used for visualization of cellular populations and NEAT1 expression.

For tissue‐level comparisons, only normal tissue and primary HNSCC samples were considered. Differential expression between the two groups was evaluated using the Wilcoxon rank‐sum test. To account for unequal numbers of cells per sample, pseudobulk expression values were calculated by averaging normalized NEAT1 expression across all cells belonging to each biological specimen. Statistical comparisons between normal tissue and primary HNSCC were performed using sample‐level pseudobulk values.

To characterize the cellular distribution of NEAT1 within tumors, analyses were restricted to primary HNSCC samples. Cell populations representing less than 5% of all primary tumor cells were excluded from the final dot plot to avoid unstable estimates arising from sparsely represented populations. Dot size represents the percentage of cells expressing NEAT1 within each cell population, whereas color intensity represents the mean normalized expression.

### 2.12 Statistical analysis

The number of biological replicates and the statistical tests applied are indicated in the corresponding figure legends. Statistical analysis was performed by using graphpad Prism (v10.2.1). Differences were considered significant for *P* < 0.05.

## 3 Results

### 3.1 NEAT1 induction upon genotoxic stresses is associated with cell survival rather than apoptosis

**Fig. 1:**
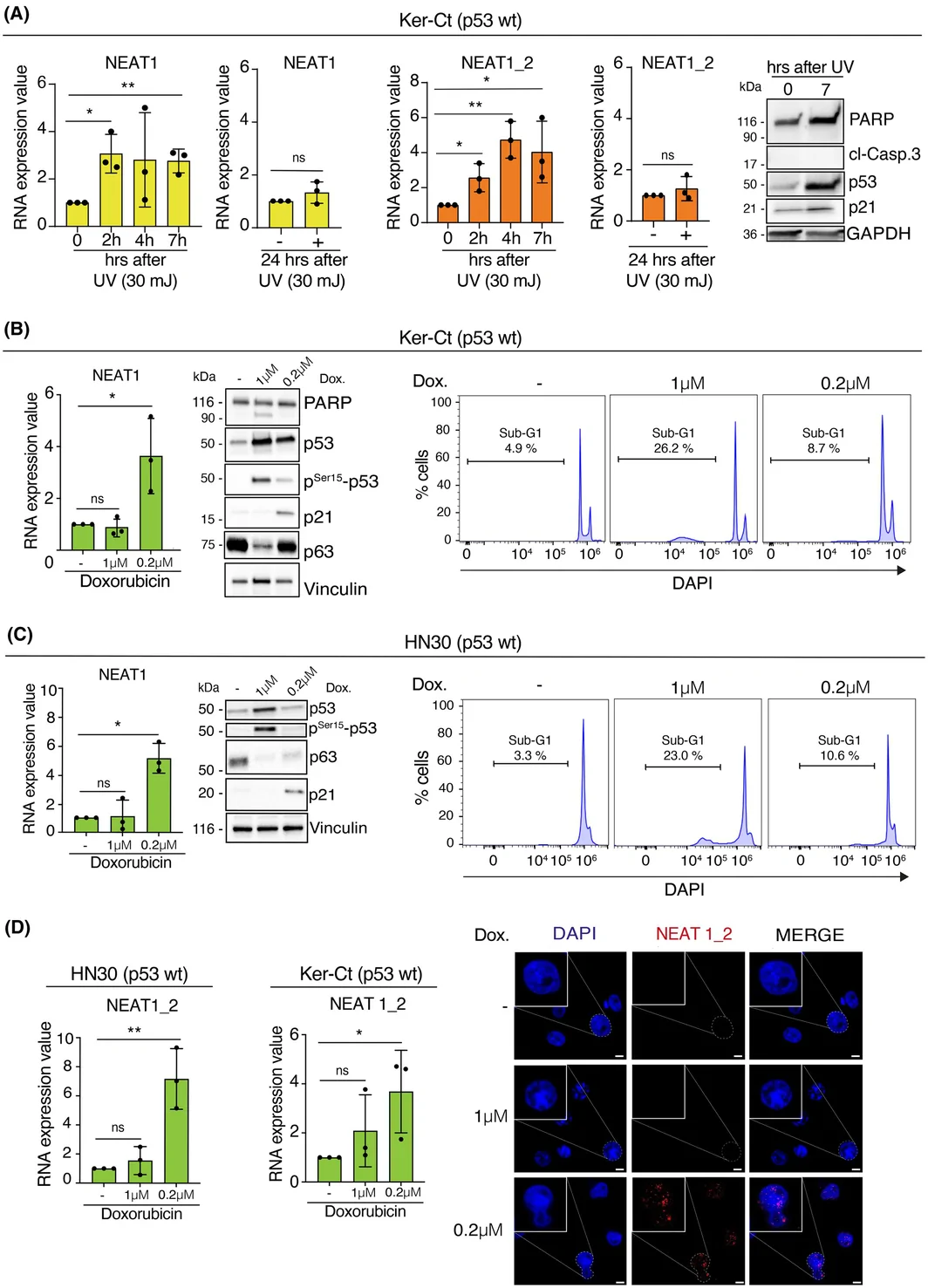
NEAT1 is induced following low doses of genotoxic stresses. (A) Human immortalized keratinocytes (Ker‐CT) were exposed to 30 mJ/cm^2^ UVB radiation. Total RNA NEAT1 (yellow) and NEAT1_2 long isoform (orange) expression levels were measured by RT‐qPCR at 2, 4, 7, and 24 h (hrs) after irradiation (left panel). Data shown are the mean of three (*n* = 3) independent biological replicates ± SD. *P* value was calculated using two‐tailed unpaired Student's *t* test: *P < 0.05; **P < 0.01; ns not significant. In parallel, the protein lysates collected at 0 and 7 h after the UVB exposure were subjected to immunoblotting (IB) using antibodies to the indicated proteins (right panel). (B) Ker‐Ct cells were treated for 18 h with 1 μm or 0.2 μm doxorubicin (dox). The levels of NEAT1 RNA and the indicated proteins were quantified by RT‐qPCR and IB, respectively, following treatment (left panel). Data shown are the mean of three (*n* = 3) independent biological replicates ± SD. *P* value was calculated using two‐tailed unpaired Student's *t* test: *P < 0.05; ns not significant. In parallel, treated cells were stained with DAPI and analyzed by flow cytometry to determine DNA content (right panel). (C) HN30 cells were treated as in (B). The levels of NEAT1 RNA and the indicated proteins were quantified by RT‐qPCR and IB, respectively, following treatment (left panel). Data shown are the mean of three (*n* = 3) independent biological replicates ± SD. *P* value was calculated using two‐tailed unpaired Student's *t* test: *P < 0.05; ns not significant. In parallel, treated cells were stained with DAPI and analyzed by flow cytometry to determine DNA content (right panel). (D) Total RNA was extracted from HN30 and Ker‐CT cells treated as in (B) and utilized to quantify the NEAT1 long isoform (NEAT1_2) RNA levels by RT‐qPCR (left panel). Data shown are the mean of three (*n* = 3) independent biological replicates ± SD. *P* value was calculated using two‐tailed unpaired Student's *t* test: *P < 0.05; **P < 0.01; ns not significant. In parallel, Ker‐CT treated cells were subjected to RNA FISH of endogenous NEAT1_2 (red). Nuclei were visualized by DAPI (blue) counterstaining (right panel). Scale bars, 10 μm. The experiment was repeated twice with similar results (*n* = 2).

Our previous studies have highlighted the critical role of the ΔNp63 in repressing NEAT1 expression in proliferating keratinocytes [24]. As NEAT1 is transcriptionally induced by p53 upon DNA damage [18, 20, 21], we analyzed NEAT1 modulation in response to genotoxic stresses in cells expressing these two transcription factors. We utilized both normal epithelial cells (Ker‐CT, immortalized human keratinocytes) and HNSCC cells (HN30) expressing ΔNp63 and wild‐type p53 (Fig. S1). As control we also used HNSCC cell lines expressing ΔNp63 and mutated p53 (FaDu, Detroit‐562, and A253) (Fig. S1). Firstly, we exposed human immortalized keratinocytes to 30 mJ/cm^2^ of UVB radiation, a type of genotoxic insult commonly occurring in the skin and known to induce a wide range of mutagenic and cytotoxic DNA lesions leading to increasing risk of skin cancer carcinogenesis [26, 27]. We measured the RNA expression levels of both total NEAT1 and the long NEAT1 isoform (NEAT1_2) by RT‐qPCR at 2‐, 4‐, 7‐, and 24‐h post‐UVB irradiation. We observed a rapid induction of NEAT1 following UVB exposure, with the peak occurring at early time points: detectable from 2 h and remaining elevated until 7 h (Fig. 1A). We observed a similar modulation also in the p53‐positive HNSCC cell line HN30 treated with UVB (Fig. S2). Notably, NEAT1 expression returned to baseline levels at 24‐h post‐treatment, indicating that its upregulation is implicated in an early response to DNA damage (Fig. 1A). Interestingly, this induction is not associated with apoptotic events, as revealed by the lack of activation of cleaved caspase‐3 and the cleavage of its substrate PARP (Fig. 1A). The absence of cleaved caspases and cleaved PARP indicates that the cells were not undergoing apoptosis in response to the UVB dose used, but probably activating a DNA damage response focused on DNA repair and cell survival. In line with this, NEAT1 induction correlated with the upregulation of p21, the cyclin‐dependent kinase inhibitor involved in cell cycle arrest (Fig. 1A). To further explore the modulation of NEAT1 in response to genotoxic stress, we exposed Ker‐CT and the p53‐positive HNSCC cell line HN30 to different doses of an additional genotoxic insult such as doxorubicin, an anthracycline antibiotic widely used in chemotherapy, able to induce DNA double‐strand breaks [28, 29]. We treated cells for 18 h with two different doses of doxorubicin, 0.2 μm or 1 μm, which exert a sublethal or lethal effect, respectively, as revealed by IB and FACS analysis (Fig. 1B,C). Interestingly, NEAT1 expression was significantly upregulated only in response to the sublethal dose of doxorubicin. This effect is associated with the induction of p21 but not with PARP cleavage (Figs 1B,C and S3). Consistently, activation of the DNA repair response was observed exclusively in cells treated with 0.2 μm of doxorubicin, as demonstrated by the upregulation of DNA repair genes, including XPC and DNA polymerase theta (POLQ) (Fig. S3). In contrast, NEAT1 was not induced in response to the higher dose of doxorubicin despite the strong activation of p53, as revealed by its phosphorylation at serine 15. Notably, we found that NEAT1_2 expression undergoes similar modulation following doxorubicin treatment (Fig. 1D, left panel). Since NEAT1_2 is required for the assembly of the paraspeckles *in vitro* and *in vivo*, we tested whether NEAT1_2 induction upon sublethal doses of doxorubicin is instrumental for the assembly of paraspeckles. As shown in Fig. 1D, the induction of NEAT1_2 expression (left panel) is associated with the assembly of paraspeckles, as revealed by the RNA fluorescence *in situ* hybridization (RNA FISH) analysis (right panel).

Altogether, these results suggest that NEAT1 modulation in response to genotoxic stresses is an early event likely associated with cell survival rather than apoptosis.

### 3.2 ΔNp63 and p53 exerts opposite functions in controlling NEAT1 expression upon genotoxic stress

**Fig. 2:**
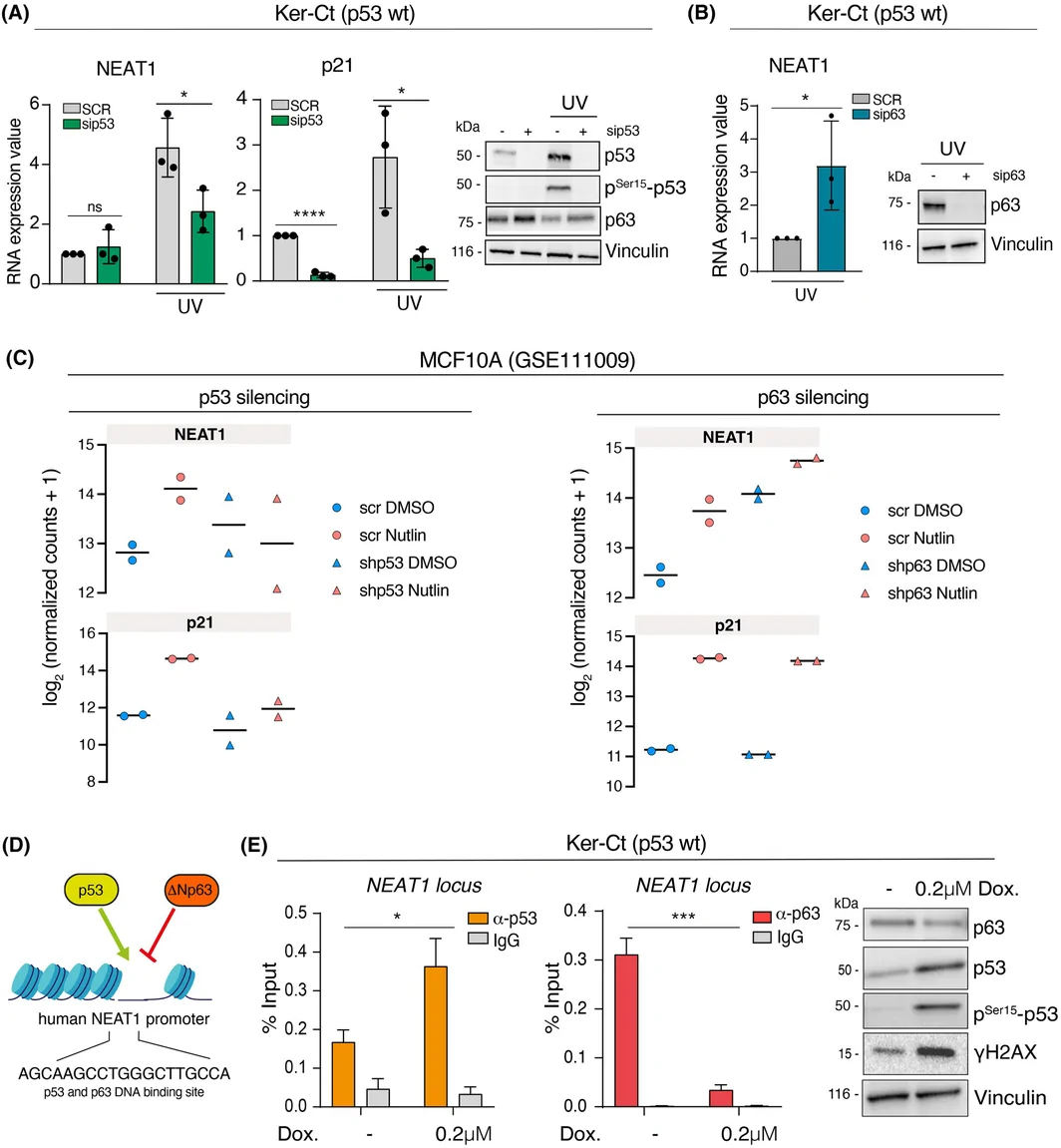
p53‐ΔNp63 interplay regulates NEAT1 expression following genotoxic stresses in HNSCC cells. (A) Ker‐CT were transfected with siRNA oligos targeting p53 (sip53) or nontargeting mRNA (SCR). NEAT1 and p21 RNA levels were quantified by RT‐qPCR (left panel). Data shown are the mean of three (*n* = 3) independent biological replicates ± SD. *P* value was calculated using two‐tailed unpaired Student's *t* test: *P < 0.05; ****P < 0.0001; ns not significant. In parallel, protein lysates from transfected cells were analyzed by IB using antibodies to the indicated proteins (right panel). (B) Ker‐CT cells were transfected with siRNA oligos targeting ΔNp63 isoform (sip63) or nonrelevant mRNA (SCR). NEAT1 RNA levels were quantified by RT‐qPCR (left panel). Data shown are the mean of three (*n* = 3) independent biological replicates ± SD. *P* value was calculated using two‐tailed unpaired Student's *t* test: *P < 0.05. In parallel, protein lysates from transfected cells were analyzed by IB using antibodies to the indicated proteins (right panel). (C) Expression of NEAT1 and p21 genes in MCF10A transfected with shRNA oligos targeting p63 (shp63), p53 (shp53) or nonrelevant mRNA (scr) treated or not with the activating p53 agent Nutlin. RNA‐seq data were downloaded from the GSE111009 RNA‐seq dataset. Data shown are the mean of two (*n* = 2) independent biological replicates. Horizontal line represents the mean value. (D) Schematic model of the regulation of NEAT1 transcription by p53‐ΔNp63 interplay. The figure was drawn using images created in Microsoft 365 PowerPoint (CNR license). (E) Representative ChIP‐qPCR experiment showing ΔNp63 and p53 occupancy at the p63/p53 binding site of *NEAT1* genomic locus following genotoxic stress (left panel). Data shown are the mean of three (*n* = 3) technical replicates ± SD. *P* value was calculated using two‐tailed unpaired Student's *t* test: *P < 0.05; ***P < 0.001. The experiment was repeated twice with similar results (*n* = 2). In parallel, protein lysates from ChIP samples were analyzed by IB using antibodies to the indicated proteins (right panel).

To investigate the role of the ΔNp63–p53 crosstalk in modulating NEAT1 expression under genotoxic stress, we first assessed whether the DNA damage‐induced upregulation of NEAT1 was dependent on p53. To this aim, human keratinocytes Ker‐CT cells were transfected with either a small interfering RNA targeting p53 (sip53) or a nontargeting scrambled control RNA (SCR). Transfected cells were then exposed to a sublethal dose of UVB radiation (30 mJ/cm^2^) and NEAT1 expression levels were evaluated by RT‐qPCR at early time points post‐treatment. As expected, p53 silencing prevented the induction of the p53 target gene p21. Similarly, NEAT1 induction upon UVB treatment is markedly impaired in p53‐silenced Ker‐CT cells (Fig. 2A). In line with these results, p53‐deficient HNSCC cells do not display any NEAT1 induction upon UVB treatment (Fig. S4), confirming the p53 dependency of NEAT1 induction upon DNA damage. To assess whether ΔNp63 still functions as a repressor of NEAT1 in UVB‐treated cells, we silenced ΔNp63 and quantified NEAT1 levels by RT‐qPCR in Ker‐CT cells exposed to UVB. We observed that ΔNp63 silencing, whose efficiency has been evaluated at RNA and protein levels (Fig. S5 and Fig. 2B), induces a significant increase in NEAT1 RNA levels (Fig. 2B). To validate this regulatory mechanism in another cellular context, we analyzed the RNA‐seq dataset GSE111009, generated from the nontransformed immortalized breast epithelial cell line MCF10A treated with the p53 activator Nutlin and subjected to p53 or p63 knockdown [30]. As shown in Fig. 2C, we confirmed in MCF10A cells that p53 and p63 exert opposing effects on NEAT1 expression following p53 activation.

Previous studies have shown that both p53 and ΔNp63 recognize the same DNA‐binding region on the *NEAT1* promoter, suggesting a potential competitive binding mechanism (Fig. 2D) [18, 24]. Based on this evidence, we investigated how DNA damage might influence the ability of these transcription factors to access the *NEAT1* promoter region. To this aim, we performed a chromatin immunoprecipitation (ChIP) assay using antibodies specific to p63 and p53 in Ker‐CT cells treated with a sublethal concentration (0.2 μm) of doxorubicin. As shown in Fig. 2E, upon DNA damage ΔNp63 occupancy at the *NEAT1* promoter markedly decreased, while p53 recruitment to the same region significantly increased. As control, we verified the activation of the DNA damage response pathways as assessed by the increase of γH2AX and p53 levels by western blot (Fig. 2E, right panel). Together, these findings support the idea that DNA damage triggers a regulatory switch between ΔNp63 and p53 at the *NEAT1* promoter. Specifically, genotoxic stress appears to displace ΔNp63 and promote the concomitant binding of p53.

### 3.3 NEAT1 targeting increases DNA damage checkpoint response in HNSCC

**Fig. 3:**
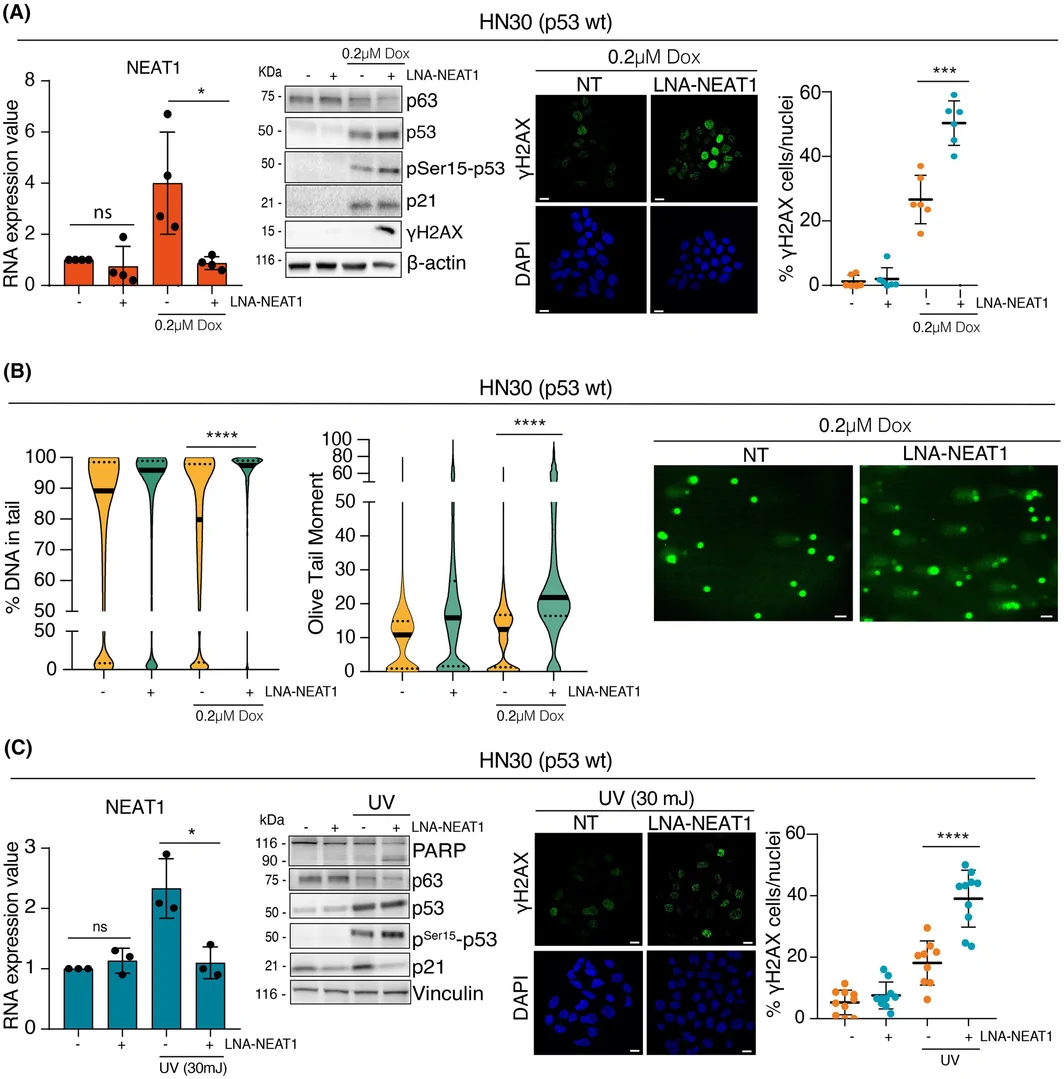
NEAT1 depletion increases DNA damage in p53‐positive HNSCC cells upon genotoxic stresses. (A) HN30 cells were transfected with negative LNA GapmeR control (NT) or LNA GapmeR NEAT1 (LNA‐NEAT1). After 48 h, cells were treated with 0.2 μm doxorubicin (dox) for 18 h. The levels of NEAT1 RNA and the indicated proteins were quantified by RT‐qPCR and IB, respectively, following treatment (left panel). Data shown are the mean of three (*n* = 3) independent biological replicates ± SD. *P* value was calculated using two‐tailed unpaired Student's *t* test: *P < 0.05; ***P < 0.001; ns not significant. In parallel, transfected cells were subjected to immunofluorescence staining performed using DAPI (blue) to visualize nuclei and γH2AX (green) to detect DNA damage. Cells with more than 5 γH2AX foci were considered positive (right panel). Scale bars, 10 μm. (B) HN30 cells transfected as in (A) were subjected to an alkaline comet assay after treatment with doxorubicin. The DNA damage was quantified using % DNA in tail and Olive tail moment parameters calculated by counting an average of 4000 nuclei derived from 2 independent biological replicates (*n* = 2). *P* value was calculated using the Mann–Whitney test: ****P < 0.0001. Representative fluorescence microscopy images of HN30 cell nuclei stained with SYBR Green (right panel). Scale bars, 50 μm. (C) HN30 cells transfected as above were exposed to 30 mJ UVB and analyzed 7‐h post‐irradiation. The levels of NEAT1 RNA and the indicated proteins were quantified by RT‐qPCR and IB, respectively, following UV treatment (left panel). Data shown are the mean of three (*n* = 3) independent biological replicates ± SD. *P* value was calculated using two‐tailed unpaired Student's *t* test: *P < 0.05; ****P < 0.0001; ns not significant. In parallel, transfected cells were subjected to immunofluorescence staining performed using DAPI (blue) and γH2AX (green), as in A) (right panel). Scale bars, 10 μm.

The results observed so far indicate that NEAT1 is rapidly induced following DNA damage, particularly at early time points and in response to sublethal concentrations of genotoxic agents. This induction is p53‐dependent and is not associated with apoptotic pathways. Based on these findings, we hypothesized that NEAT1 may play a functional role in the DNA damage response pathway, particularly during the early stages when cells are still viable and have not yet committed to irreversible cell fate decisions such as apoptosis. To test this hypothesis, we conducted loss‐of‐function experiments by silencing NEAT1 using a mix of LNA‐gapmer antisense oligonucleotides in p53‐expressing HN30 cells treated with 0.2 μm of doxorubicin. As shown in Fig. 3A, NEAT1 depletion induces a marked increase in the levels of γH2AX, a well‐established marker of DNA double‐strand breaks and an early indicator of DNA damage signaling, and an increase in p53 activation. Similar results were obtained also in Ker‐CT cells (Fig. S6). To further validate these observations, we measured the extent of the DNA damage at the single‐cell level by performing the comet assay in NEAT1‐depleted HN30 cells upon doxorubicin treatment. As shown in Fig. 3B, NEAT1 depletion significantly increases the extent of DNA damage in individual cells.

To validate these findings with an additional DNA‐damaging agent, we exposed HN30 cells to 30 mJ/cm^2^ of UVB radiation following NEAT1 knockdown and assessed DNA damage at the single‐cell level by performing γH2AX immunofluorescence. Consistently, immunofluorescence analysis revealed a significant increase in the number of γH2AX‐positive cells in NEAT1‐depleted cells upon UVB insults (Fig. 3C). Together, these findings strongly suggest that NEAT1 helps to maintain genomic stability during early stress responses and its loss impairs DNA damage resolution.

### 3.4 NEAT1 silencing enhances givinostat cytotoxicity in TP53‐mutated HNSCC cell lines

**Fig. 4:**
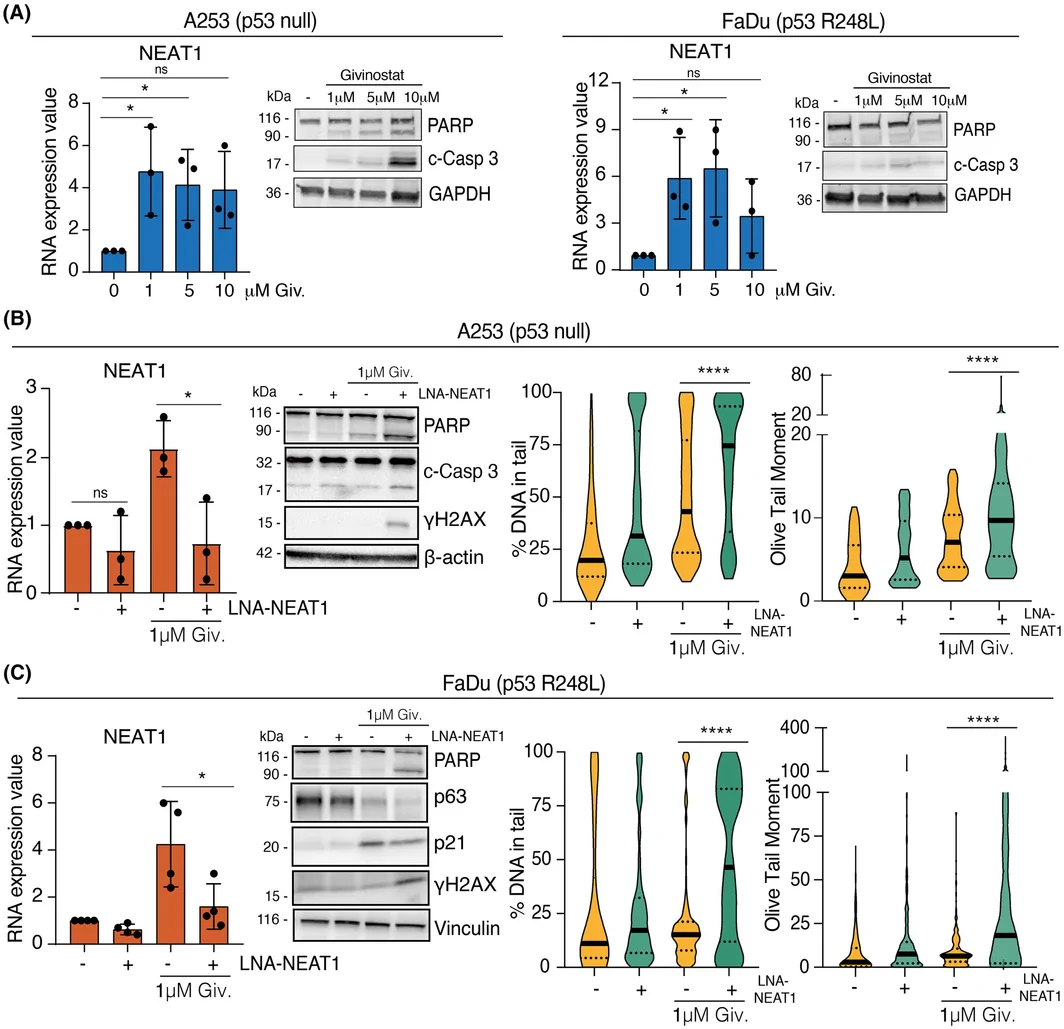
NEAT1 targeting increases the cytotoxic effects of HDAC1/2 inhibitor givinostat in HNSCC cells defective of p53 function. (A) A253 and FaDu cells were treated with the indicated doses of the HDAC1/2 inhibitor givinostat. 18‐h post‐treatment, NEAT1 RNA levels were quantified by RT‐qPCR (left panel). Data shown are the mean of three (*n* = 3) independent biological replicates ± SD. *P* value was calculated using two‐tailed unpaired Student's *t* test: *P < 0.05; ns not significant. In parallel, protein lysates extracted from givinostat‐treated cells were analyzed by IB using antibodies to the indicated proteins (right panel). (B) A253 cells were transfected with negative LNA GapmeR control (−) or LNA GapmeR NEAT1 (LNA‐NEAT1). After 48 h, cells were treated with 1 μm givinostat for 24 h. The levels of NEAT1 RNA and the indicated proteins were quantified by RT‐qPCR and IB, respectively, following treatment (left panel). Data shown are the mean of three (*n* = 3) independent biological replicates ± SD. *P* value was calculated using two‐tailed unpaired Student's *t* test. *P < 0.05; ****P < 0.0001; ns not significant. In parallel, cell pellets form transfected cells were subjected to an alkaline comet assay to quantify DNA damage using % DNA in tail and Olive tail moment parameters (right panel) calculated by counting nuclei derived from 2 independent biological replicates (*n* = 2). *P* value was calculated using the Mann–Whitney test. (C) FaDu cells were transfected as above and exposed to 1 μm givinostat for 24 h. The levels of NEAT1 RNA and the indicated proteins were quantified by RT‐qPCR and IB, respectively, following givinostat treatment (left panel). Data shown are the mean of three (*n* = 3) independent biological replicates ± SD. *P* value was calculated using two‐tailed unpaired Student's *t* test. In parallel, cell pellets form transfected cells were subjected to an alkaline comet assay to quantify DNA damage using % DNA in Tail and Olive tail moment parameters (right panel) calculated by counting nuclei derived from 2 independent biological replicates (*n* = 2). *P* value was calculated using the Mann–Whitney test: *P < 0.05; ****P < 0.0001.

The results presented so far suggest that genotoxic insults induce NEAT1 expression in a p53‐dependent manner and that NEAT1 may play a role in modulating the DNA damage response, raising the possibility that targeting NEAT1 could enhance the cytotoxic effects of chemotherapeutic agents. However, since the majority of SCC tumors harbor TP53 mutations, this approach may have limited applicability. Our previous study demonstrated that givinostat, a class I and II HDAC inhibitor currently approved as the first non‐steroidal drug for Duchenne muscular dystrophy [31, 32, 33], induces a marked increase in NEAT1 levels in human primary keratinocytes [24]. Based on this observation, we aimed to investigate whether NEAT1 targeting might impact the givinostat‐mediated cellular response in p53‐mutated cell lines, provided that HDAC inhibitors also regulate NEAT1 expression in *TP53* mutated tumor cells. Therefore, as a first step, we investigated the effect of givinostat treatment on NEAT1 RNA levels in *TP53* mutated HNSCC cell lines. In detail, we treated A253 (p53 null) and FaDu (p53 R248L mutation) cells with increasing concentrations of givinostat. RT‐qPCR analysis of NEAT1 RNA levels revealed a consistent and marked upregulation of NEAT1 in both cell lines (Fig. 4A). We observed a marked increase of NEAT1 upregulation also in HNSCC cells treated with an additional pan HDAC inhibitor (panobinostat) or selective HDAC class 1 inhibitor (entinostat) (Fig. S7). These data indicate that givinostat‐mediated NEAT1 induction occurs independently of p53 status, thus opening the possibility to study the givinostat‐NEAT1 axis in p53 mutated HNSCC cells. To this aim, we first conducted experiments to establish the appropriate experimental conditions for this study. A253 and FaDu HNSCC cell lines were treated with increasing concentrations of givinostat, and the apoptotic profile analyzed by western blot and FACS analysis. We observed a dose‐dependent increase of PARP cleavage and activation of caspase 3 in A253 cells, while FaDu cells seem to be more resistant to givinostat cytotoxic effects (Fig. 4A). Additionally, in both cell types givinostat treatment induces the dose‐dependent accumulation of cells in the G2/M phase of cell cycle, suggesting cell cycle arrest likely due to DNA damage (Fig. S8).

Importantly, NEAT1 was already strongly induced at 1 μm givinostat, a concentration that did not trigger significant apoptosis in A253 cells. Based on this, we selected 1 μm of givinostat as working dose for assessing the specific contribution of NEAT1 to givinostat's cytotoxic effects, as it allows NEAT1 induction without excessive cell death. Therefore, we transfected A253 cells with LNA‐GapmeR targeting NEAT1 and measured the extent of DNA damage and the cytotoxicity effects upon givinostat treatment. We observed that NEAT1 silencing leads to an increase in the cytotoxic effects of givinostat, as revealed by the increase in the levels of γH2AX, cleaved caspase‐3, sub‐G1 population and decrease in the colony forming efficiency (Fig. 4B left panel, and Fig. S9). Along the same line, we observed an increase in the DNA damage by comet assay in NEAT1‐depleted cells upon givinostat treatment (Fig. 4B, right panel). Similarly, NEAT1 silencing resulted in an increase in DNA damage also in FaDu cells treated with givinostat (Fig. 4C). To extend these findings to murine SCC models, we first analyzed NEAT1 expression upon givinostat treatment in two mouse SCC cell lines, SCC7 and MOC1. As shown in Fig. S10A, givinostat induces a significant upregulation of NEAT1 in these murine SCC cells, indicating that NEAT1 regulation in response to HDAC inhibition is evolutionarily conserved. Importantly, NEAT1 knockdown using a combination of LNA gapmers enhanced givinostat‐induced DNA damage in SCC7 cells, as demonstrated by the Comet assay. In line with this, we found that depletion of NEAT1 also significantly impairs the clonogenic potential of these cells (Fig. S10B).

These results suggest that NEAT1 depletion enhances givinostat's cytotoxic efficacy, supporting a potential therapeutic strategy of combining NEAT1 targeting with HDAC inhibitors to improve treatment outcomes in HNSCC, particularly in the p53‐mutated genetic context.

### 3.5 NEAT1 expression is increased in HNSCC patients

**Fig. 5:**
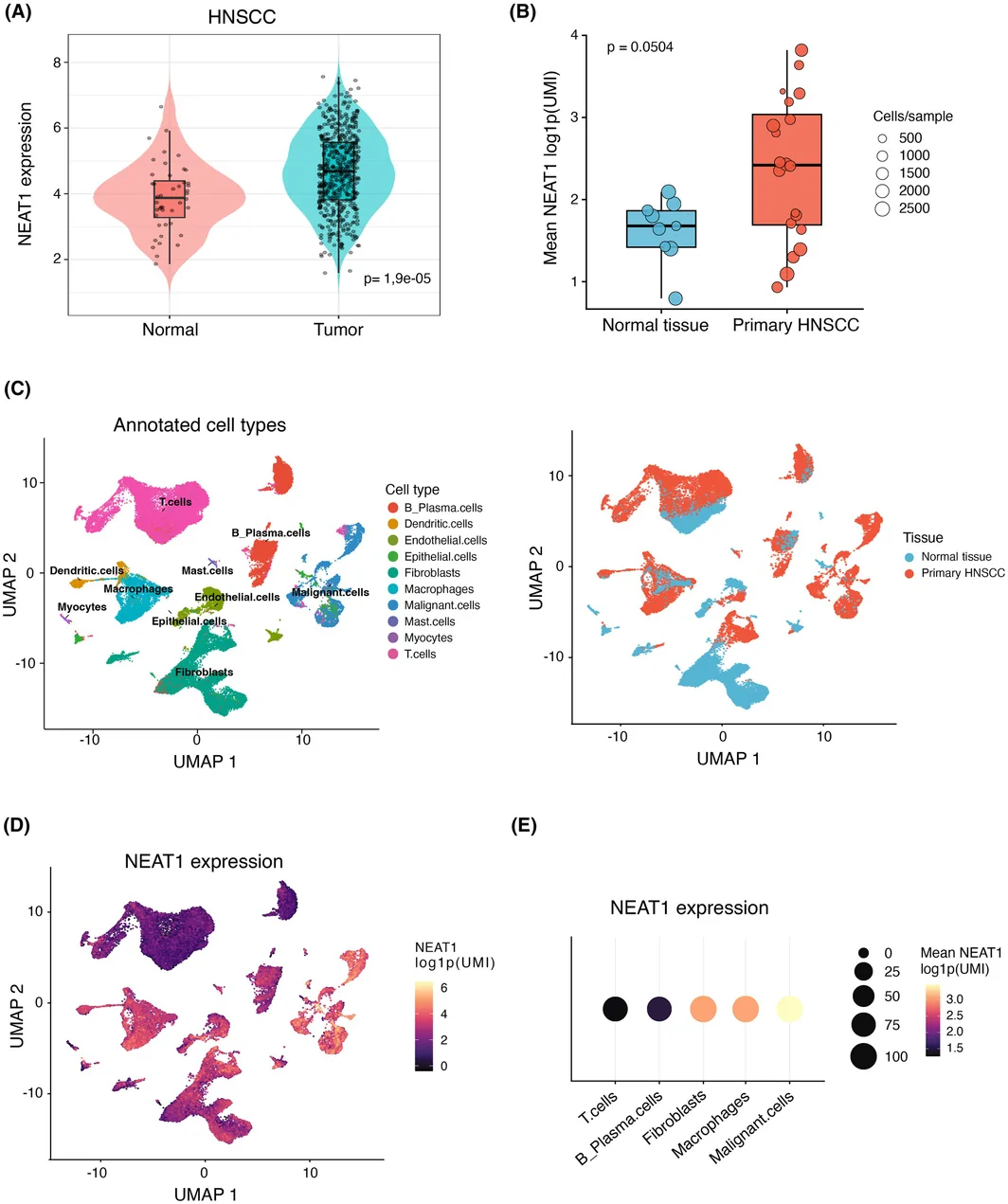
NEAT1 expression is increased in HNSCC samples. (A) Violin plot illustrating NEAT1 expression values in HNSCC (tumor *n* = 522, normal = 44) from the TCGA dataset. (B–E) single‐cell NEAT1 expression in normal oral tissue and primary HNSCC cells from the GSE181919 dataset. (B) Pseudobulk analysis of NEAT1 expression across biological samples. For each sample, normalized NEAT1 expression was calculated by averaging log1p‐transformed UMI counts across all cells. Each dot represents a biological sample, and dot size is proportional to the number of cells contributing to each sample. Statistical significance was assessed using the Wilcoxon rank‐sum test. (C) UMAP representation of all annotated cell populations (left) and tissue origin (right). Cell identities were assigned according to the original dataset annotation. The right panel highlights the distribution of cells originating from normal tissue and primary HNSCC. (D) UMAP showing NEAT1 expression across all cells. Color intensity represents normalized log1p‐transformed UMI counts. (E) Dot plot summarizing NEAT1 expression across major cell populations in primary HNSCC samples. Dot color indicates the average normalized NEAT1 expression, whereas dot size represents the percentage of cells expressing NEAT1 within each population. Only cell populations accounting for at least 5% of all primary HNSCC cells were included.

The regulation of NEAT1 expression in human tumors is highly context dependent. In general, NEAT1 is upregulated in many solid tumors, where it exerts oncogenic functions [22]. However, in certain malignancies, such as acute promyelocytic leukemia, NEAT1 expression is downregulated, likely reflecting its role in promoting cellular differentiation [21, 34]. To investigate NEAT1 expression in SCC, we analyzed data from The Cancer Genome Atlas (TCGA), comparing tumor samples with matched normal tissues. This analysis revealed that NEAT1 expression is significantly increased in HNSCC patients, whereas no significant differences were observed in lung and esophageal SCCs (Figs 5A and S11A). We further examined whether NEAT1 expression varies across HNSCC subtypes. However, no significant differences were detected according to HPV status, molecular subtype, or anatomical site (Fig. S11B). To further confirm these findings, we evaluated *NEAT1* expression in normal oral mucosa and primary HNSCC tissues using the scRNA‐seq dataset GSE181919 (Fig. 5B–E). Pseudobulk analysis showed an upregulation of NEAT1 in HNSCC tissues compared to healthy controls (Fig. 5B). Furthermore, cell‐type‐specific analysis revealed that malignant cells exhibit the highest expression levels among primary tumor‐derived cell subsets (Fig. 5E).

## 4 Discussion

NEAT1 is a long noncoding RNA which has been initially characterized as an essential RNA of the ribonucleoprotein structures called paraspeckles. Subsequent studies have delineated NEAT1 as a multifunctional regulatory RNA involved in multiple signaling pathways [16]. Our group has previously demonstrated that NEAT1 plays a crucial role in modulating squamous differentiation [24]. In detail, we showed that in proliferating keratinocytes the epithelial transcription factor ΔNp63 represses NEAT1 transcription by an HDAC‐dependent mechanism. Upon the activation of epidermal differentiation, the release of the ΔNp63‐mediated repression of NEAT1 allows NEAT1 accumulation which in turn modulates the expression of epidermal differentiation markers. In the present study, we investigated the role of NEAT1 in HNSCC, a tumor type characterized by genetic alterations affecting genes involved in the regulation of the squamous differentiation program, including ΔNp63. Based on previous studies demonstrating that p53 transcriptionally regulates NEAT1 in response to DNA damage [18, 21], we first explored how the interplay between p53 and ΔNp63 regulates NEAT1 expression. We found that NEAT1 upregulation upon genotoxic stress was dependent on p53 activity and was further enhanced by ΔNp63 silencing, highlighting the functional antagonism between these two transcription factors. Consistently, ChIP experiments demonstrated that DNA damage promotes ΔNp63 dissociation and p53 recruitment to the *NEAT1* promoter region, thereby enabling NEAT1 transcriptional activation. This dynamic ‘chromatin switch’ can result from different molecular mechanisms. Since ΔNp63 undergoes ubiquitin‐dependent degradation upon DNA damage, it is possible that ΔNp63 degradation facilitates p53 binding to the *NEAT1* promoter. In line with this, we observed a slight reduction of ΔNp63 protein levels following treatment with low doses of doxorubicin. Alternatively, phosphorylation of ΔNp63 at specific residues by kinases involved in the DNA damage response, including ATM, CDK2, and p70S6K [35, 36] may reduce its affinity for chromatin, thus favoring p53‐dependent NEAT1 induction. Regardless of the precise mechanism, our findings are consistent with accumulating evidence identifying NEAT1 as a critical component of the cellular stress response. For instance, under mitochondrial stress the transcription factor ATF2 induces NEAT1 expression, promoting paraspeckle assembly, and the retention of specific mRNAs involved in mitochondrial repair [37]. Under hypoxic conditions, HIF‐2α transcriptionally activates NEAT1, which in turn stabilizes HIF complexes and enhances hypoxia‐responsive gene expression [38]. Similarly, during heat shock, HSF1‐mediated NEAT1 induction supports an efficient cellular response to proteotoxic stress [39]. Overall, these data clearly pinpoint NEAT1 as a central lncRNA orchestrating adaptive responses to multiple forms of cellular stress.

Several lines of evidence have indicated that NEAT1 modulation upon DNA damage is instrumental to regulate the DNA damage response (DDR). Upon replicative stress, NEAT1 promotes ATR signaling and its depletion results in increased DNA damage accumulation [18]. In multiple myeloma and ovarian cancer cells, NEAT1 depletion increases DNA damage and sensitizes cells to PARP inhibitors [19, 40, 41]. In line with these observations, we found that NEAT1 silencing in p53‐positive cells exposed to sublethal doses of doxorubicin led to increased γH2AX accumulation, indicating impaired DDR and higher levels of DNA damage. Additionally, NEAT1‐deficient cells displayed enhanced activation of p53, reflecting higher cellular stress compared to controls. Although the molecular mechanisms underlying NEAT1‐dependent regulation of the DDR were not directly investigated in this study, several hypotheses can be proposed. NEAT1 may regulate the transcription of DDR‐related genes by recruiting transcriptional regulators to their promoters. This possibility is supported by studies showing that NEAT1 depletion in multiple myeloma and ovarian cancer cells reduces the expression of key homologous recombination factors, including RAD51, CHK1/2, RPA32, and BRCA1 [19, 41]. Alternatively, NEAT1 might affect DDR indirectly as an indispensable part of paraspeckles. Several paraspeckle‐associated proteins, including NONO, SFPQ, and RBM14, are known to participate in DNA repair pathways. NONO and SFPQ contribute to both NHEJ and HR through interactions with proteins such as KU70/KU80, RAD51, and TOPBP1 [42, 43]. Additional paraspeckle‐associated proteins, including FUS/TLS and BRG1, also support homologous recombination and genomic stability. The involvement of paraspeckle components in the DDR raises the possibility that NEAT1 itself localizes at sites of DNA damage. In agreement with this hypothesis, METTL3‐mediated m6A methylation of NEAT1 has been shown to promote its accumulation at double‐strand breaks and facilitate recruitment of repair proteins such as CHD4 [44]. However, other studies have reported that paraspeckles and their associated proteins do not colocalize with DNA damage foci [18], suggesting that paraspeckles may instead regulate DDR indirectly, for example, by modulating the availability and localization of DNA repair factors. In this context, paraspeckles may function as dynamic hubs capable of retaining or releasing RNA‐binding proteins and repair‐associated factors according to cellular needs.

Although the molecular details through which NEAT1 regulates the DDR remain to be fully elucidated, the available evidence provides proof‐of‐principle that targeting NEAT1 may enhance the cytotoxic effects of genotoxic therapies. However, many human tumors, including a large fraction of HNSCC, lack functional p53 activity and therefore fail to induce NEAT1 in response to genotoxic insults. Interestingly, we found that class I/II HDAC inhibitors act as potent inducers of NEAT1 expression across SCC cell lines, independently of p53 status. These observations suggest that NEAT1 upregulation may represent part of the broader adaptive response to the transcriptional and replicative stress induced by HDAC inhibition. Accordingly, we found that NEAT1‐silenced cells exposed to givinostat exhibited increased DNA damage, as indicated by alkaline comet assays and γH2AX staining. Notably, this enhanced sensitivity was observed in both p53 wild‐type and p53‐mutant SCC cells. These findings suggest that NEAT1 inhibition may potentiate the antitumor efficacy of HDAC inhibitors independently of p53 status, thus opening new therapeutic perspectives based on the combination of HDAC inhibitors with lncRNA‐targeting strategies. It is worth noting that the HDACI/II inhibitor givinostat has been recently approved by the U.S. Food and Drug Administration (FDA) as the first nonsteroidal treatment for Duchenne muscular dystrophy [31]. Moreover, several clinical trials evaluating the antitumor activity of HDAC inhibitors, either alone or in combination with conventional chemotherapeutic agents, are currently ongoing [33].

In conclusion, our findings suggest that combining NEAT1 targeting with HDAC inhibition may represent a promising therapeutic strategy to enhance treatment efficacy, particularly in *TP53*‐mutant HNSCCs.

## 5 Conclusions

In this study, we demonstrate that in HNSCC the functional interplay between p53 and ΔNp63 regulates NEAT1 expression in response to genotoxic stress. At a functional level, we show that targeting NEAT1 could be therapeutically exploited to enhance the cytotoxic effects of anti‐neoplastic drugs, particularly HDAC1/2 inhibitors, which induce NEAT1 transcription independently of p53 status. Future investigations are required to dissect the molecular mechanisms underlying NEAT1‐dependent signaling in the DNA damage response.

## Conflict of interest

The authors have no conflicts of interest to declare.

## Author contributions

AP conceived the project. SDD, VLB, SDA, and SN performed all experiments. SS performed the bioinformatic analysis. SDD prepared the figures. AP and SN wrote the paper. All the authors revised the manuscript and approved the final version.

## Supporting information


**Fig. S1.** p53 and p63 protein levels in HNSCC cell lines. Representative immunoblotting (IB) analysis of the protein lysates extracted from human immortalized keratinocytes (Ker‐CT) and HNSCC cell lines (HN30, FaDu, Detroit‐562, A253) using antibodies to the indicated proteins. The experiment was repeated twice with similar results (*n* = 2).
**Fig. S2.** NEAT1 expression in HN30 cells exposed to UVB radiation. HN30 cells were exposed to 30 mJ/cm^2^ UVB radiation. After 7 h RNA was extracted and levels of NEAT1 and NEAT1_2 long isoform were measured by RT‐qPCR at the indicated time points. Data shown are the mean of three (n = 3) independent biological replicates ± SD. *P* value was calculated using two‐tailed unpaired Student's *t* test: * *P* < 0.05.
**Fig. S3.** Expression of *POLQ*, *XPC* and *p21* genes in Ker‐CT and HN30 cells upon genotoxic stresses. Ker‐CT (top) and HN30 (bottom) cells were treated with the indicated doses of Doxorubicin for 18 h. RNA levels of the indicated genes were quantified by RT‐qPCR. Data shown are the mean of three (*n* = 3) independent biological replicates ± SD. *P* value was calculated using two‐tailed unpaired Student's *t* test: *P < 0.05; **P < 0.01.
**Fig. S4.** NEAT1 expression in A253 and FaDu cells exposed to UVB radiation. The indicated HNSCC cell lines were exposed to 30 mJ/cm^2^ UVB radiation. At the indicated time points, total RNA was extracted and utilized to quantify NEAT1 and NEAT1_2 long isoform RNA levels by RT‐qPCR. Data shown are the mean of three (*n* = 3) independent biological replicates ± SD. ns: not significant.
**Fig. S5.** p63 silencing efficiency in Ker‐CT cells exposed to UVB radiation. Ker‐CT cells were transfected with siRNA oligos targeting ΔNp63 isoform (sip63) or non‐relevant mRNA (SCR). p63 RNA levels were quantified by RT‐qPCR. Data shown are the mean of three (*n* = 3) independent biological replicates ± SD. *P* value was calculated using two‐tailed unpaired Student's *t* test: *P < 0.05.
**Fig. S6.** NEAT1 depletion increases DNA damage in Ker‐CT cells upon genotoxic stresses. Ker‐CT cells were transfected with non‐targeting (NT) or NEAT1‐targeting LNA GapmeRs (LNA‐NEAT1). After 48 h, cells were treated with 0.2 μm doxorubicin for 18 h. NEAT1 expression was measured by RT‐qPCR (left panel). Data shown are the mean of two (*n* = 2) independent biological replicates. In parallel, the levels of the indicated proteins were analyzed by IB (right panel).
**Fig. S7.** HDAC inhibitors increase NEAT1 expression in A253 cells. A253 HNSCC cells were treated for 18 h with the indicated concentration of the selective HDAC class 1 inhibitor (entinostat) or with the pan HDAC inhibitor (panobinostat). NEAT1 RNA levels were quantified by RT‐qPCR. Data shown are the mean of four (*n* = 4) or three (*n* = 3) independent biological replicates ± SD. *P* value was calculated using two‐tailed unpaired Student's *t* test: * *P* < 0.05; **P < 0.01; ***P < 0.001; ns not significant.
**Fig. S8.** Givinostat alters cell cycle distribution in p53‐mutant HNSCC cells. Cell cycle profiles of A253, and FaDu cells at 18 h post‐treatment with the indicated doses of givinostat (*n* = 1 biological replicate). Cells were stained with propidium iodide and analyzed by flow cytometry to determine DNA content and distribution across the cell cycle phases.
**Fig. S9.** NEAT1 targeting increases the cytotoxic effects of the HDAC1/2 inhibitor givinostat in A253 cells. A253 cells (p53 null) were transfected with non‐targeting (NT) or NEAT1‐targeting LNA GapmeRs (LNA‐NEAT1). After 48 h, cells were treated with 1 μm givinostat for 18 h. Cells were stained with propidium iodide and analyzed by flow cytometry to determine DNA content and distribution across the cell cycle phases (left panel). In parallel, transfected cells were plated at low density to perform colony forming assay (right panel). The experiment was repeated twice with similar results (*n* = 2 biological replicates).
**Fig. S10.** NEAT1 targeting increases the cytotoxic effects of the HDAC1/2 inhibitor givinostat in murine SCC7 cells. (A) SCC7 and MOC1 cells were treated for 18 h with the indicated doses of Givinostat. NEAT1 RNA levels were quantified by RT‐qPCR. Data shown are the mean of three (*n* = 3) independent biological replicates ± SD. *P* value was calculated using two‐tailed unpaired Student's *t* test: * *P* < 0.05; **P < 0.01. (B) SCC7 cells were transfected with negative LNA GapmeR control (−) or LNA GapmeR NEAT1 (LNA‐NEAT1) and after 48 h, cells were treated with 1 μm givinostat for 24 h. Cell pellets were subjected to an alkaline comet assay to quantify DNA damage using % DNA in tail and Olive tail moment parameters (top panel) calculated by counting single nuclei derived from two independent biological replicates (*n* = 2). *P* value was calculated using the Mann–Whitney test: ****P < 0.0001. NEAT1 levels were quantified by RT‐qPCR following treatment (bottom left panel). Data shown are the mean of two (*n* = 2) independent biological replicates. In parallel, transfected cells were also plated at low density to perform colony forming assay (bottom right panel).
**Fig. S11.** NEAT1 expression across SCC subtypes. (A) Violin plot illustrating NEAT1 expression values in lung SCC (LUSC, *n* = 501, normal = 51) and esophageal SCC (ESCA, *n* = 96, normal = 13). (B) Violin plot illustrating NEAT1 expression values in the indicated molecular and anatomical subtypes of HNSCC samples. *P* value was calculated using two‐tailed unpaired Student's *t* test.
**Fig. S12.** Uncropped images related to the blots/gels shown in the Figures.
**Fig. S13.** Uncropped images related to the blots/gels shown in the Figures.

## Data Availability

Data that support the findings of this study are available from the corresponding author (angelo.peschiaroli@cnr.it) upon reasonable request.
