# Supplementary material for: Regulation of the lncRNA NEAT1 by p53‐ΔNp63 crosstalk modulates the DNA damage response and therapeutic efficacy in HNSCC
Source: Mol Oncol. 2026 Sep 7:10.1002/1878-0261.70331. Online ahead of print. doi: 10.1002/1878-0261.70331 (PMC13548822; doi:10.1002/1878-0261.70331)
Supplement: Supplementary file 1 [file MOL2-9999-0-s001.pdf]

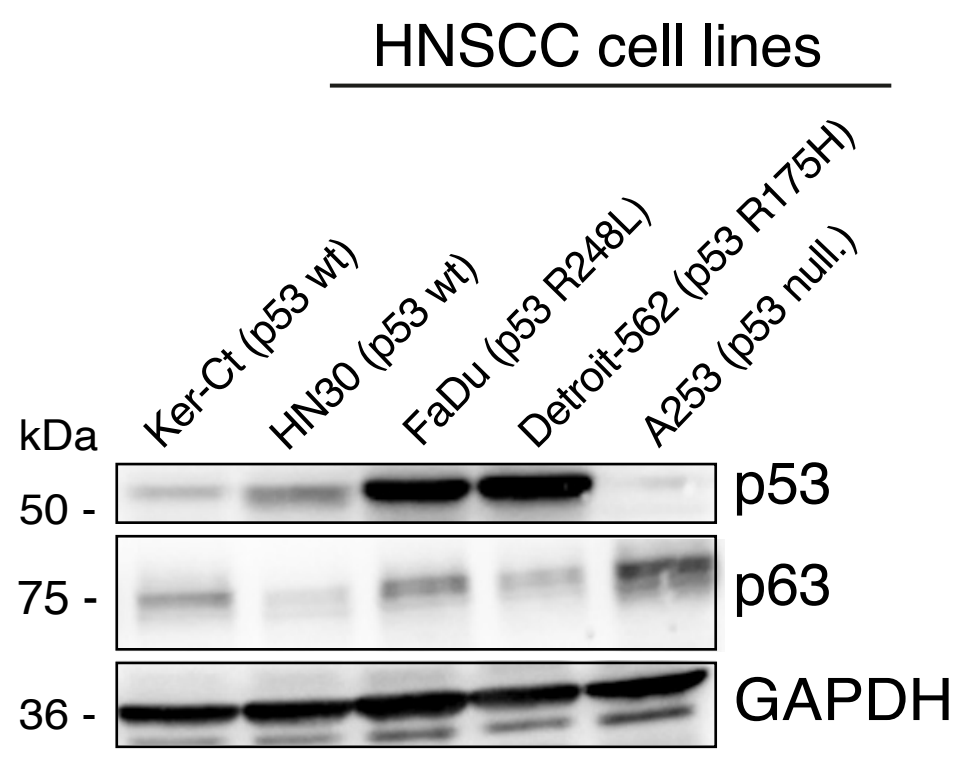

Figure S1. **p53 and p63 protein levels in HNSCC cell lines**. Representative immunoblotting (IB) analysis of the protein lysates extracted from human immortalized keratinocytes (Ker-CT) and HNSCC cell lines (HN30, FaDu, Detroit-562, A253) using antibodies to the indicated proteins. The experiment was repeated twice with similar results (n=2).

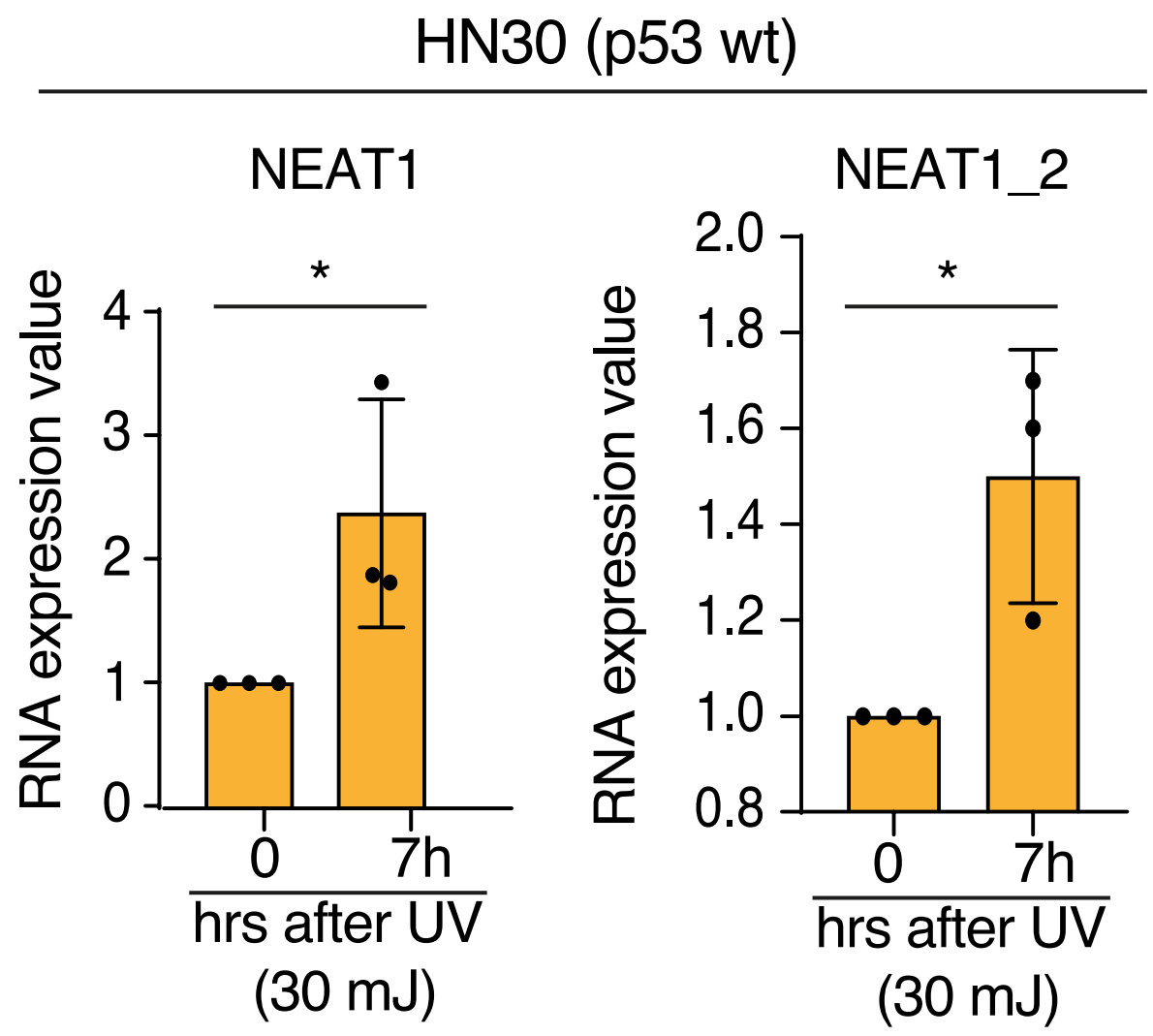

Figure S2. NEAT1 expression in HN30 cells exposed to UVB radiation. HN30 cells were exposed to 30 mJ/cm² UVB radiation. After 7hrs RNA was extracted and levels of NEAT1 and NEAT1_2 long isoform were measured by RT-qPCR at the indicated time points. Data shown are the mean of three (n=3) independent biological replicates ± SD. p value was calculated using two-tailed unpaired Student's t test: * $p < 0.05$.

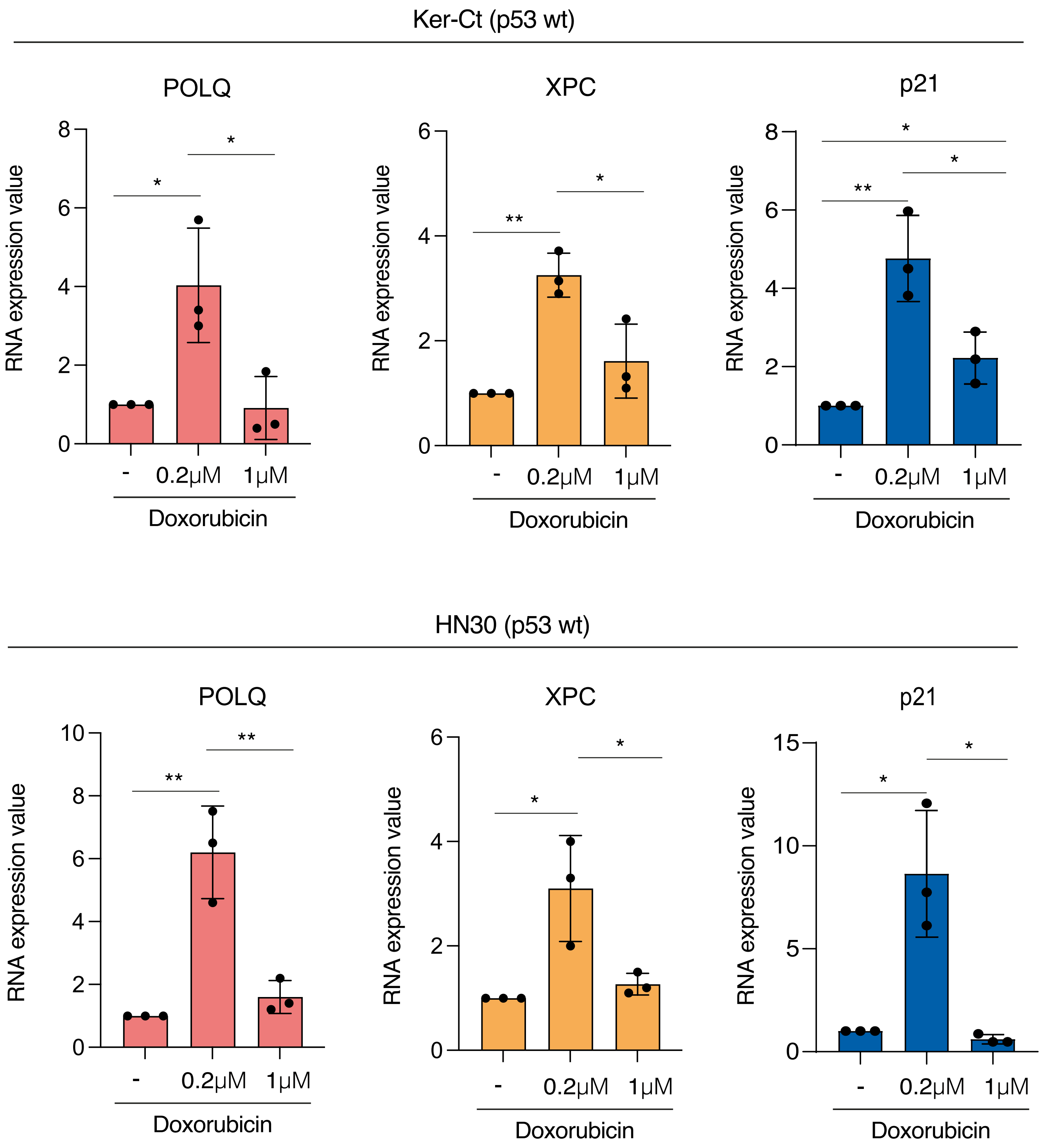

Figure S3. **Expression of *POLQ*, *XPC* and *p21* genes in Ker-CT and HN30 cells upon genotoxic stresses.** Ker-CT (top) and HN30 (bottom) cells were treated with the indicated doses of Doxorubicin for 18 hours. RNA levels of the indicated genes were quantified by RT-qPCR. Data shown are the mean of three (n=3) independent biological replicates ± SD. p value was calculated using two-tailed unpaired Student's t test: * $p < 0.05$; ** $p < 0.01$.

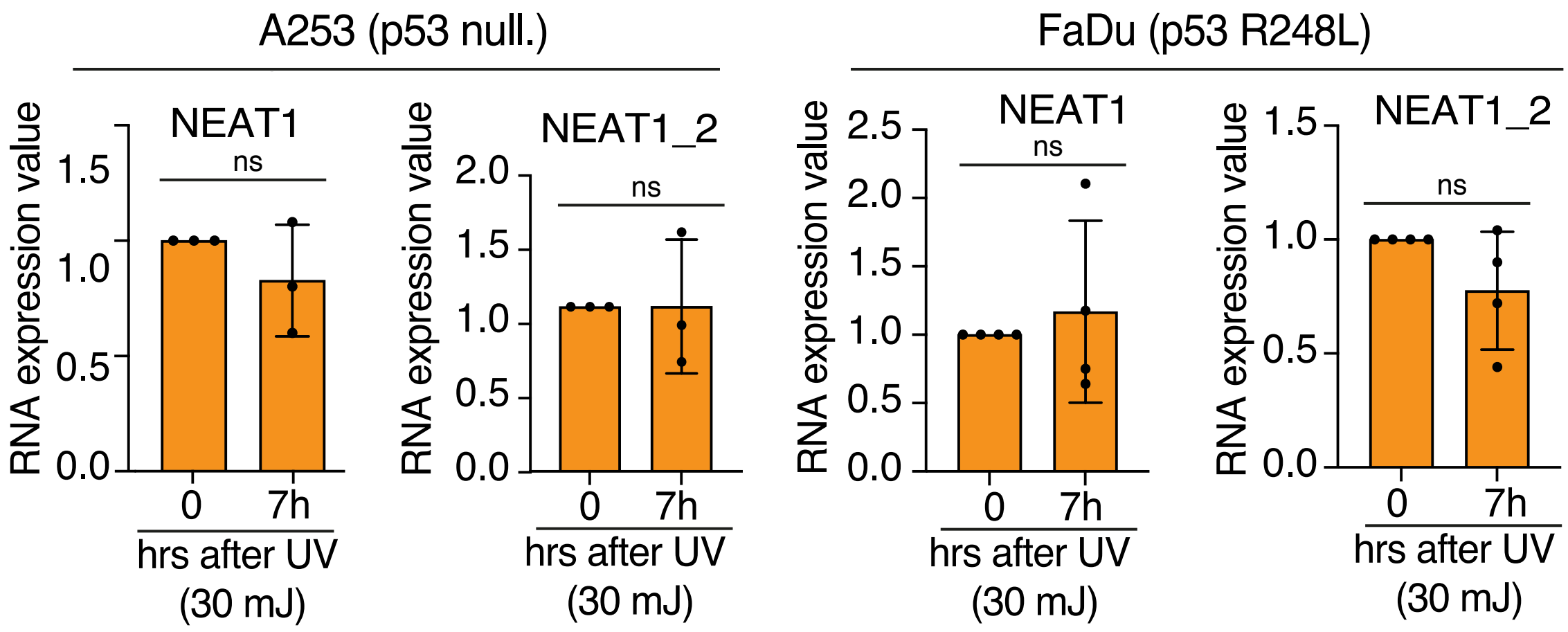

Figure S4. NEAT1 expression in A253 and FaDu cells exposed to UVB radiation. The indicated HNSCC cell lines were exposed to 30 mJ/cm² UVB radiation. At the indicated time points, total RNA was extracted and utilized to quantify NEAT1 and NEAT1_2 long isoform RNA levels by RT-qPCR. Data shown are the mean of three (n=3) independent biological replicates ± SD. ns: not significant.

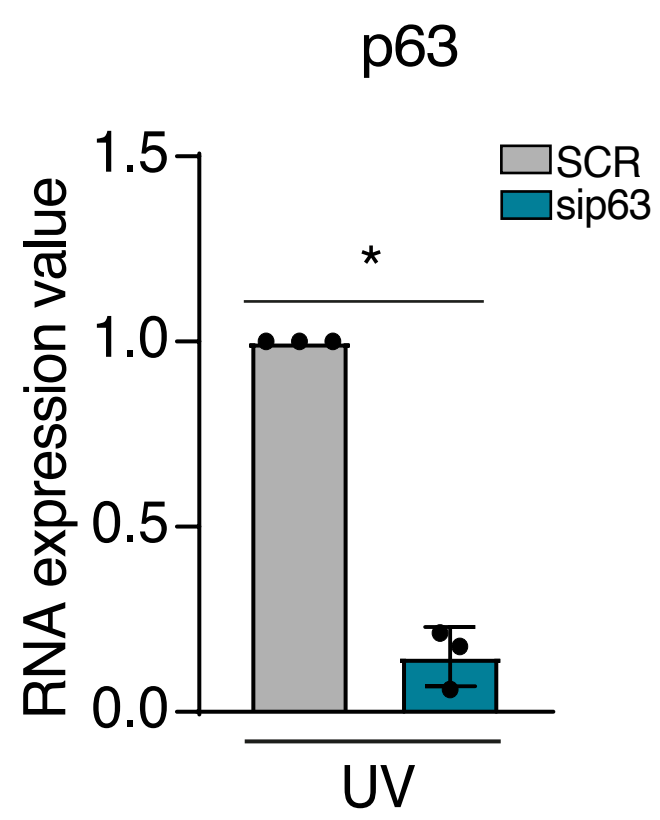

Figure S5. p63 silencing efficiency in Ker-CT cells exposed to UVB radiation. Ker-CT cells were transfected with siRNA oligos targeting ΔNp63 isoform (sip63) or non-relevant mRNA (SCR). p63 RNA levels were quantified by RT-qPCR. Data shown are the mean of three (n=3) independent biological replicates ± SD. p value was calculated using two-tailed unpaired Student's t test: * $p < 0.05$.

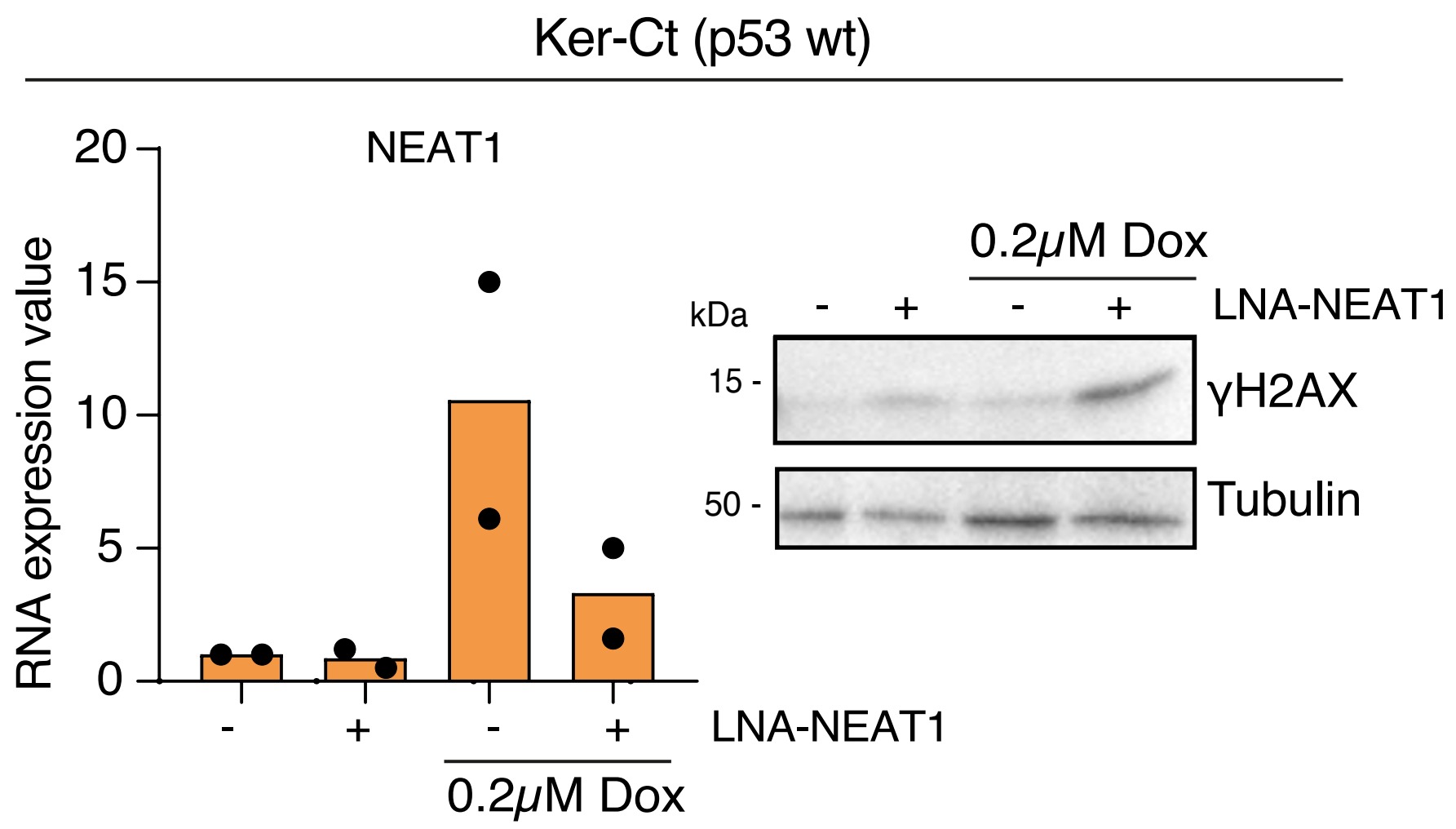

Figure S6. NEAT1 depletion increases DNA damage in Ker-CT cells upon genotoxic stresses. Ker-CT cells were transfected with non-targeting (NT) or NEAT1-targeting LNA-GapmeRs (LNA-NEAT1). After 48 hrs, cells were treated with 0.2 µM doxorubicin for 18 hrs. NEAT1 expression was measured by RT-qPCR (left panel). Data shown are the mean of two (n=2) independent biological replicates. In parallel, the levels of the indicated proteins were analyzed by IB (right panel).

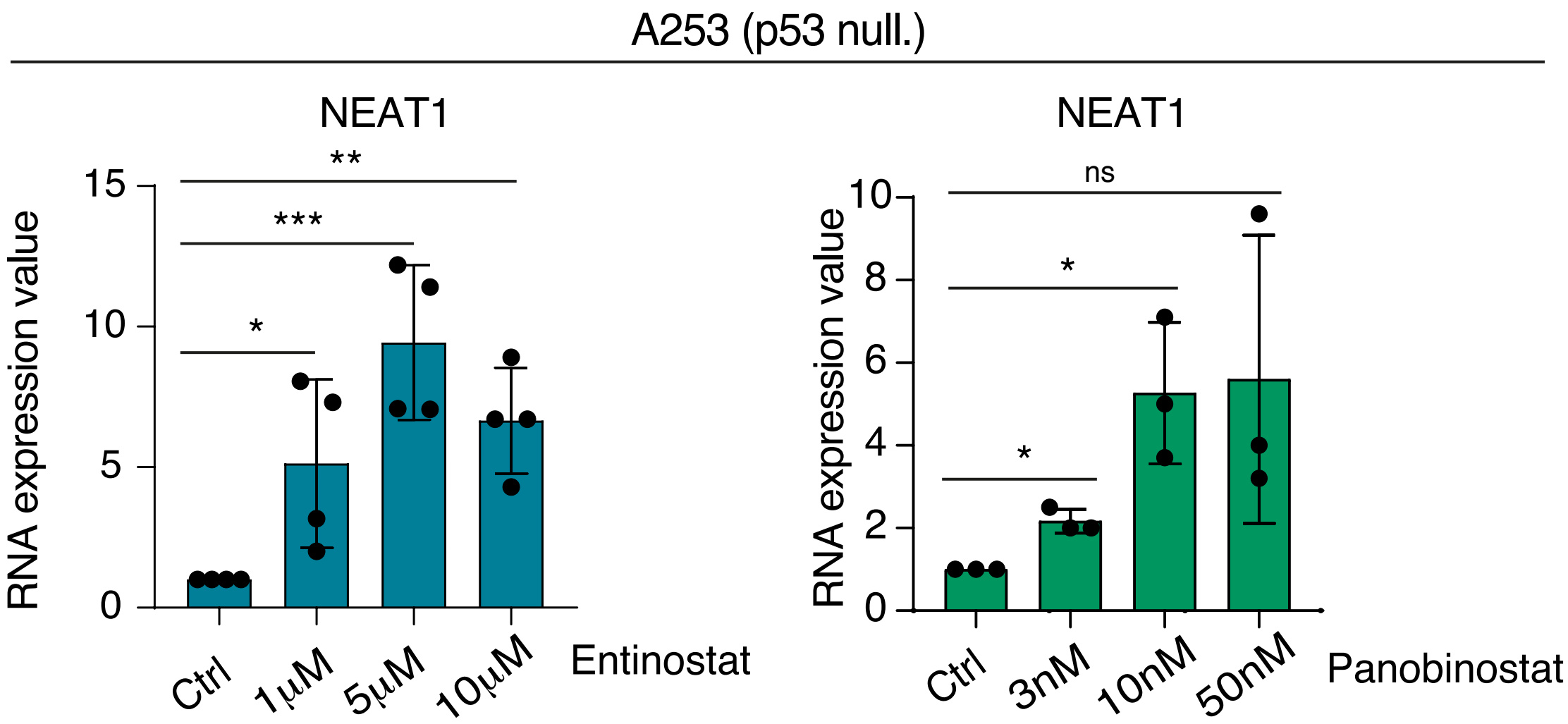

Figure S7. HDAC inhibitors increase NEAT1 expression in A253 cells. A253 HNSCC cells were treated for 18 hours with the indicated concentration of the selective HDAC class 1 inibitor (entinostat) or with the pan HDAC inhibitor (panobinostat). NEAT1 RNA levels were quantified by RT-qPCR. Data shown are the mean of four (n=4) or three (n=3) independent biological replicates ± SD. p value was calculated using two-tailed unpaired Student's t test: * $p < 0.05$; ** $p < 0.01$; *** $p < 0.001$; ns not significant.

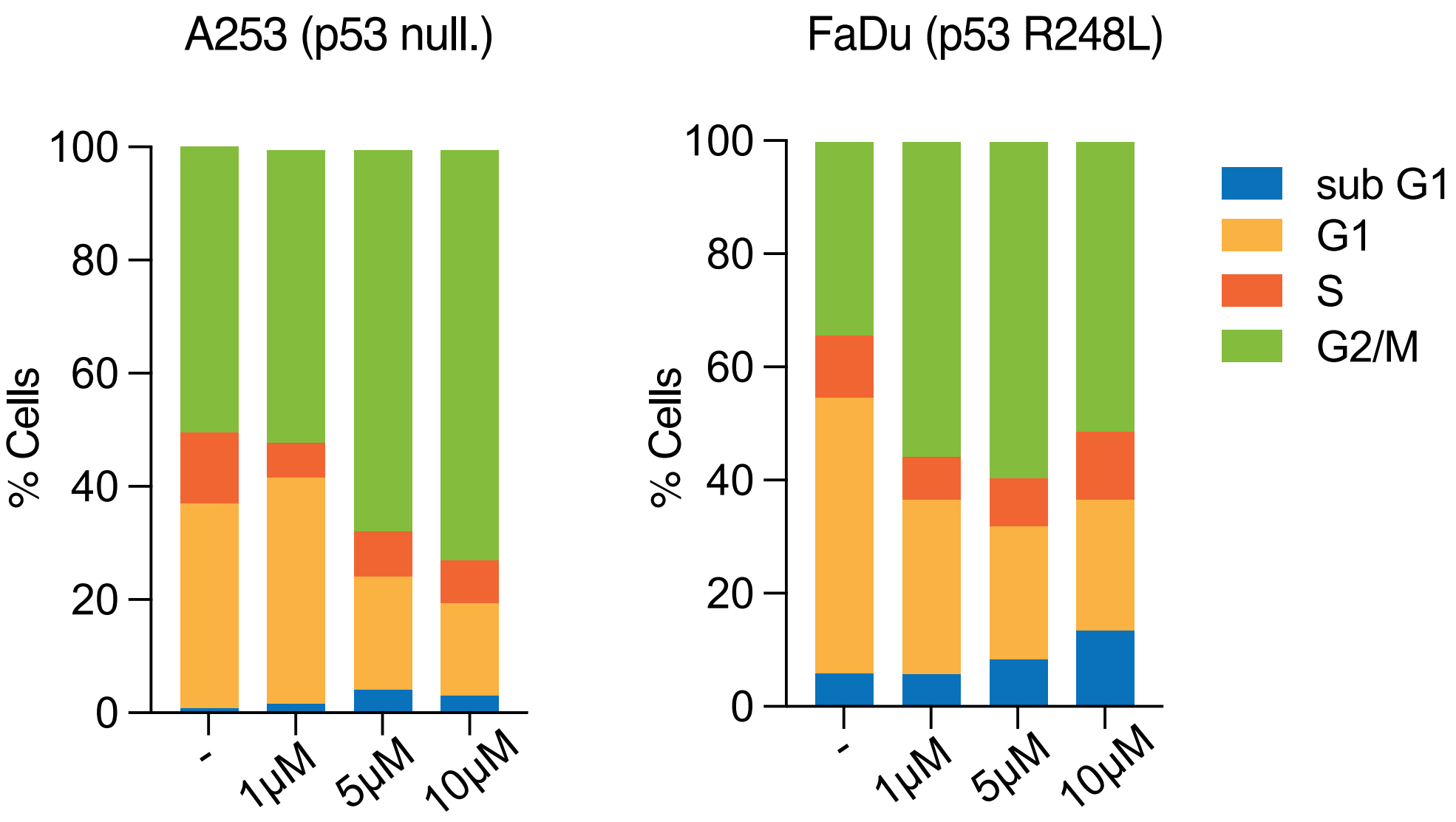

Figure S8. Givinostat alters cell cycle distribution in p53-mutant HNSCC cells. Cell cycle profiles of A253, and FaDu cells at 18 hours post-treatment with the indicated doses of givinostat (n=1 biological replicate). Cells were stained with propidium iodide and analyzed by flow cytometry to determine DNA content and distribution across the cell cycle phases.

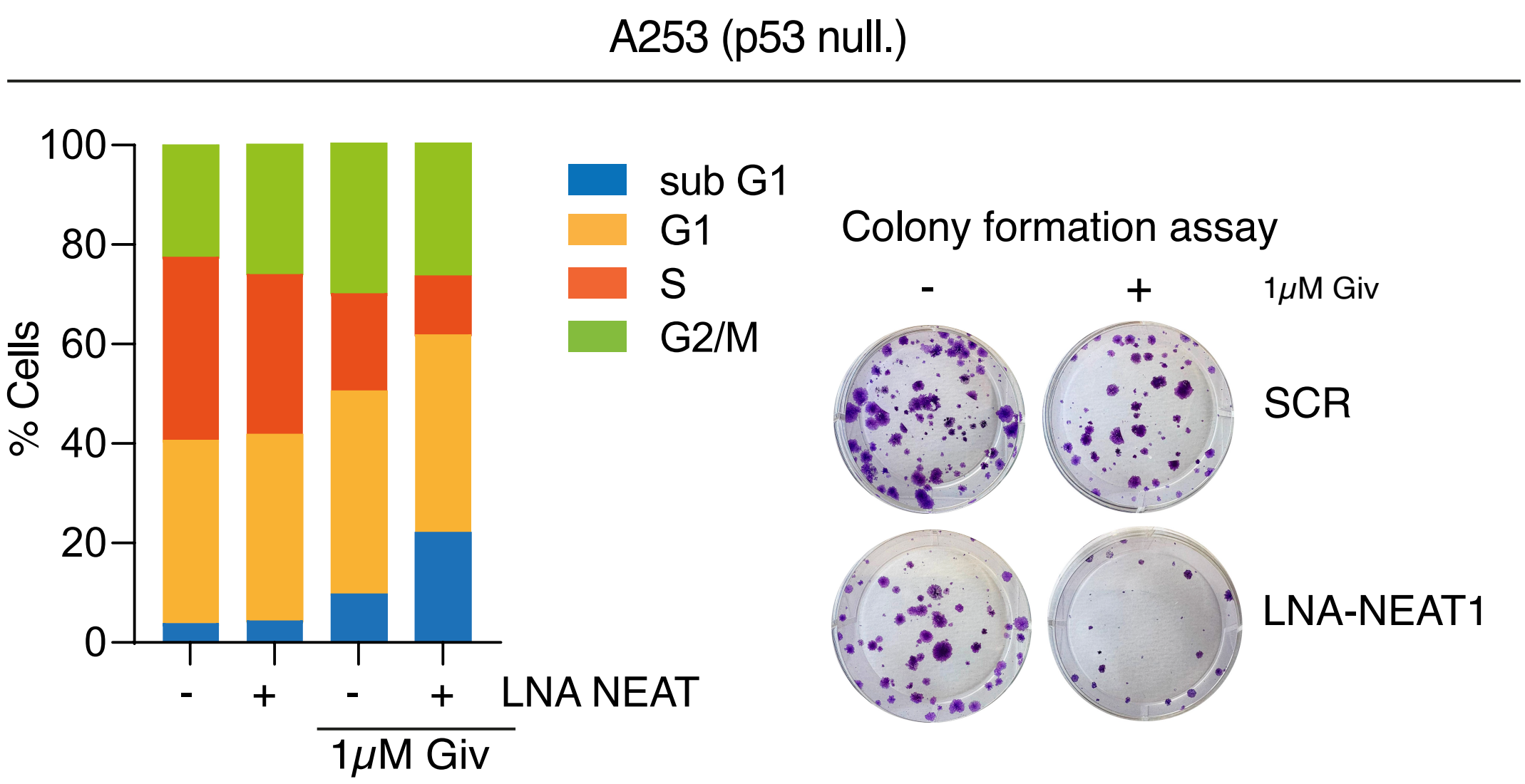

Figure S9. NEAT1 targeting increases the cytotoxic effects of the HDAC1/2 inhibitor givinostat in A253 cells. A253 cells (p53 null) were transfected with non-targeting (NT) or NEAT1-targeting LNA-GapmeRs (LNA-NEAT1). After 48 hrs, cells were treated with 1µM givinostat for 18 hrs. Cells were stained with propidium iodide and analyzed by flow cytometry to determine DNA content and distribution across the cell cycle phases (left panel). In parallel, transfected cells were plated at low density to perform colony forming assay (right panel). The experiment was repeated twice with similar results (n=2 biological replicates).

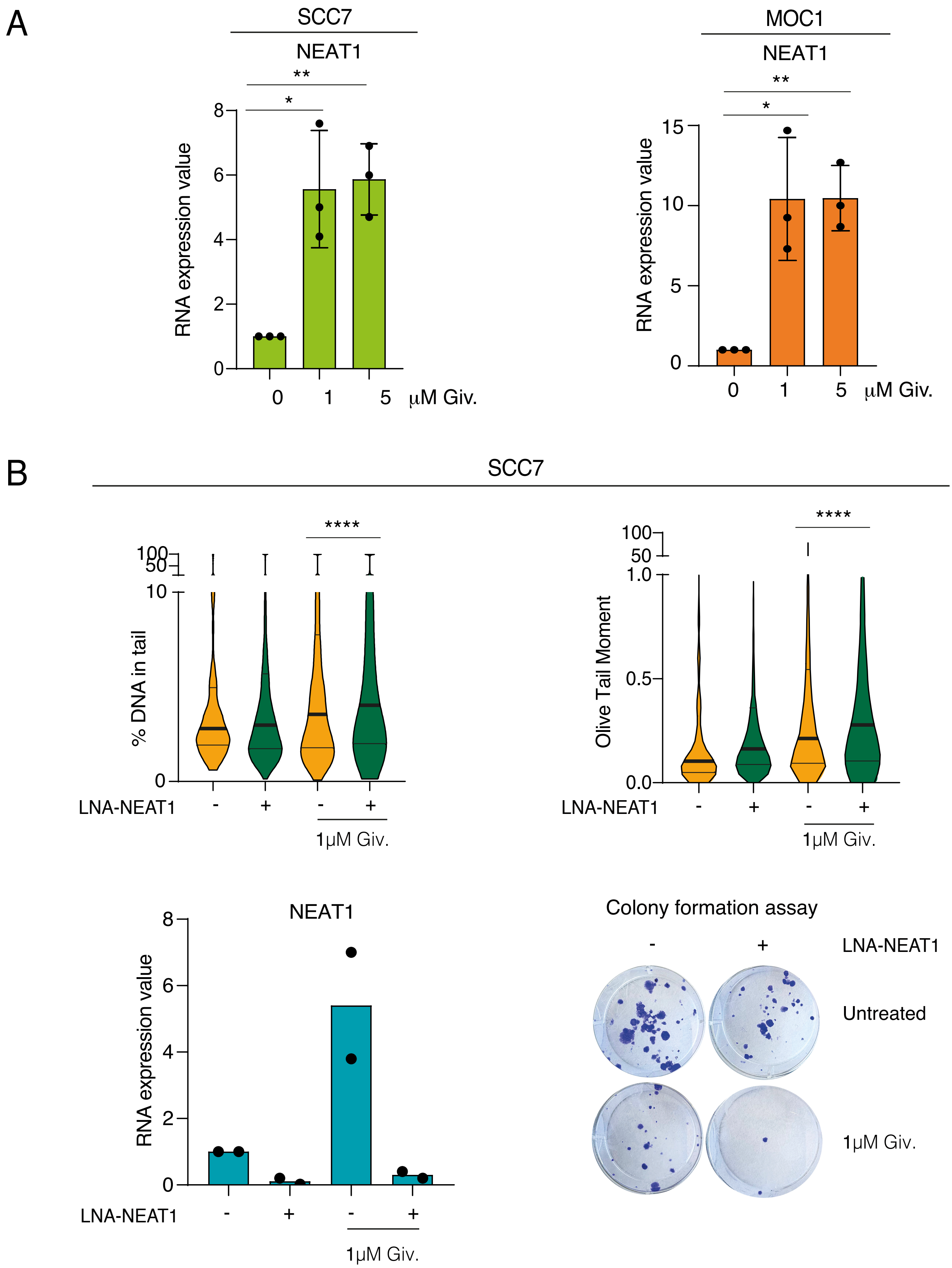

Figure S10. NEAT1 targeting increases the cytotoxic effects of the HDAC1/2 inhibitor givinostat in murine SCC7 cells. A) SCC7 and MOC1 cells were treated for 18 hours with the indicated doses of Givinostat. NEAT1 RNA levels were quantified by RT-qPCR. Data shown are the mean of three (n=3) independent biological replicates ± SD. p value was calculated using two-tailed unpaired Student's t test: * $p < 0.05$; ** $p < 0.01$. B) SCC7 cells were transfected with negative LNA GapmeR control (-) or LNA GapmeR NEAT1 (LNA-NEAT1) and after 48 hrs, cells were treated with 1 µM givinostat for 24 hrs. Cell pellets were subjected to an alkaline comet assay to quantify DNA damage using % DNA in tail and Olive tail moment parameters (top panel) calculated by counting single nuclei derived from two independent biological replicates (n=2). p value was calculated using the Mann–Whitney test: **** $p < 0.0001$. NEAT1 levels were quantified by RT-qPCR following treatment (bottom left panel). Data shown are the mean of two (n=2) independent biological replicates. In parallel, transfected cells were also plated at low density to perform colony forming assay (bottom right panel).

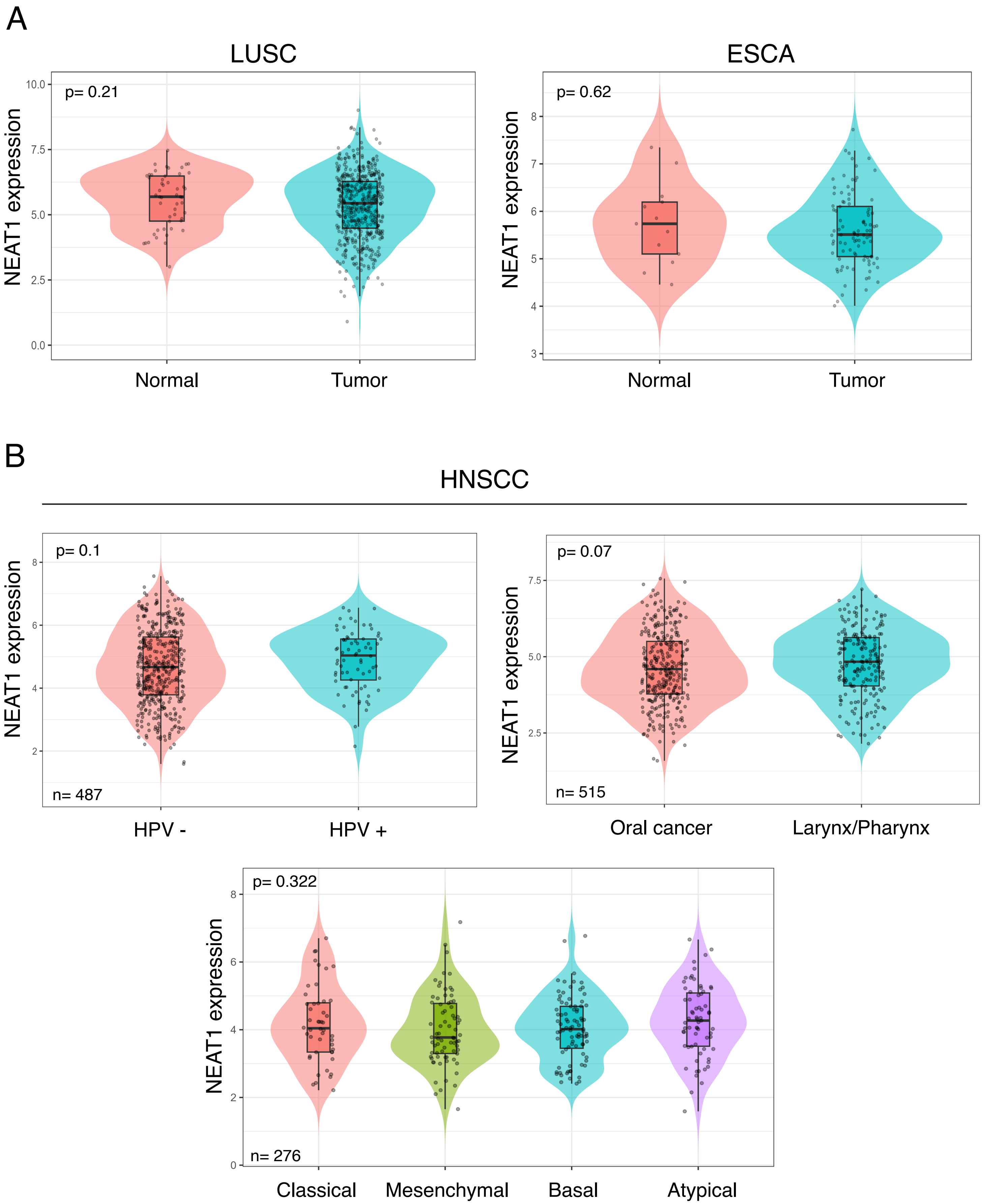

Figure S11. NEAT1 expression across SCC subtypes. A) Violin plot illustrating NEAT1 expression values in lung SCC (LUSC, n=501, normal=51) and esophageal SCC (ESCA, n=96, normal=13). B) Violin plot illustrating NEAT1 expression values in the indicated molecular and anatomical subtypes of HNSCC samples. p value was calculated using two-tailed unpaired Student's t test.

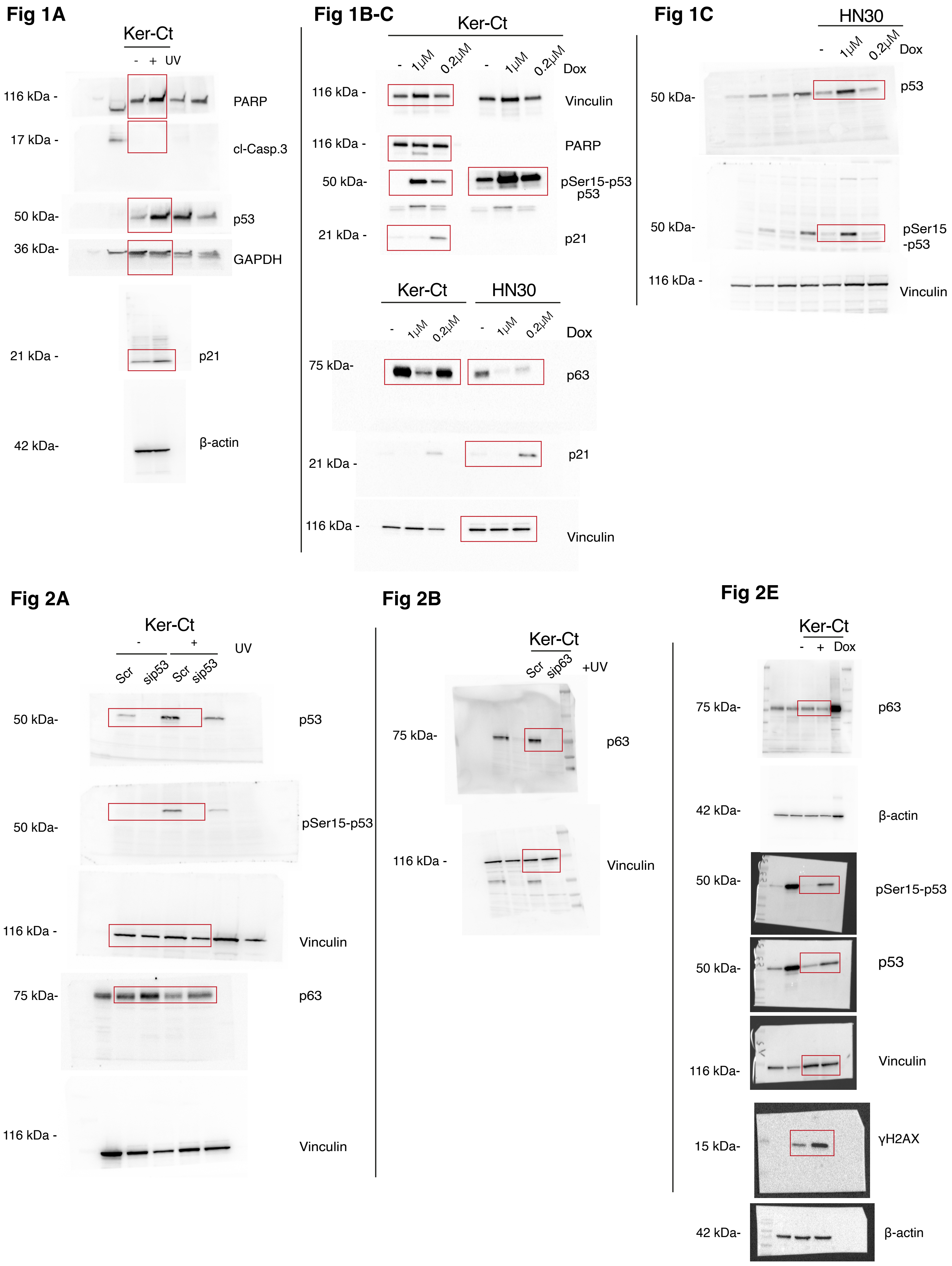

Figure S12. Uncropped images related to the blots/gels shown in the Figures.

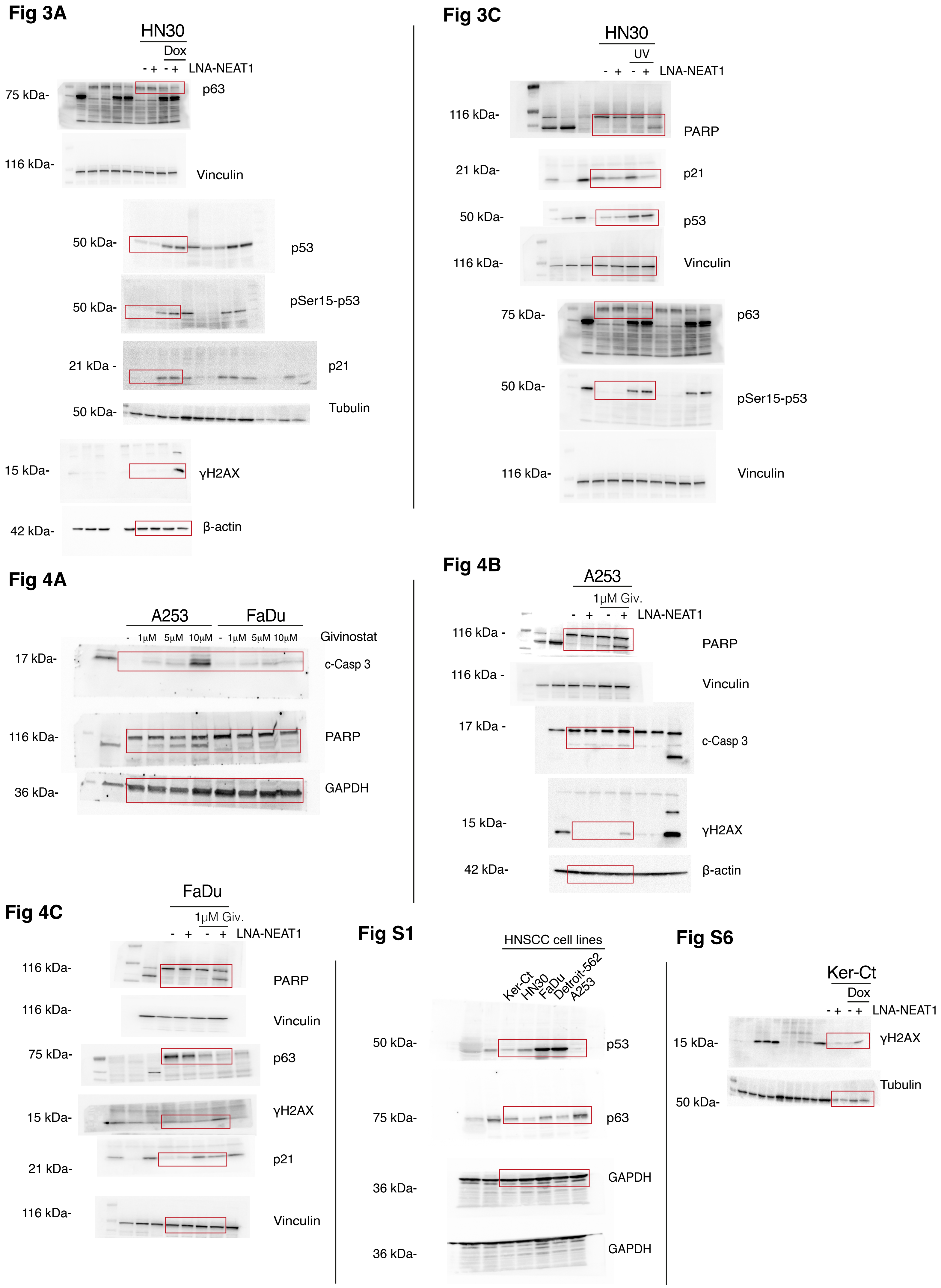

Figure S13. Uncropped images related to the blots/gels shown in the Figures.
